# Solid-state self-template synthesis of Ta-doped Li_2_ZnTi_3_O_8_ spheres for efficient and durable lithium storage

**DOI:** 10.1016/j.isci.2021.102991

**Published:** 2021-08-18

**Authors:** Dongwei Ma, Jiahui Li, Jing Yang, Chengfu Yang, Maykel Manawan, Yongri Liang, Ting Feng, Yong-Wei Zhang, Jia Hong Pan

**Affiliations:** 1 MOE Key Laboratory of Resources and Environmental Systems Optimization, College of Environmental Science and Engineering, North China Electric Power University, Beijing 102206, China; 2 Institute of High Performance Computing, Agency for Science, Technology and Research (A∗STAR), 1 Fusionopolis Way, #16-16 Connexis, Singapore 138632, Singapore; 3 Fakultas Teknologi Pertahanan, Universitas Pertahanan Indonesia, Jawa Barat 16810, Indonesia; 4 State Key Lab of Metastable Materials Science and Technology, and School of Materials Science and Engineering, Yanshan University, Qinhuangdao 066012, Hebei, China; 5 School of Metallurgical and Ecological Engineering, University of Science & Technology Beijing, Beijing 100083, China

**Keywords:** Energy systems, Energy storage, Materials synthesis, Energy materials

## Abstract

Ta-doped Li_2_ZnTi_3_O_8_ (LZTO) spheres (Li_2_ZnTi_3-*x*_Ta_*x*_O_8_; where *x* is the synthetic chemical input, *x* = 0, 0.03, 0.05, 0.07) are synthesized via solid-state reaction using mesoporous TiO_2_ spheres as the self-template. The majority of Ta^5+^ ions are uniformly doped into crystal lattices of LZTO through the Ti↔Ta substitution, and the rest forms the piezoelectric LiTaO_3_ secondary phase on the surface, as confirmed by X-ray diffraction refinement, Raman spectroscopy, density functional theory, and electron microscopy. Electrochemical impedance spectroscopy demonstrates that the Ta^5+^ doping creates rapid electronic transportation channels for high Li^+^ ion diffusion kinetics; however, the LiTaO_3_ surface coating is beneficial to improve the electronic conductivity. At the optimal *x* = 0.05, Li_2_ZnTi_3-*x*_Ta_*x*_O_8_ spheres exhibit a reversible capacity of 90.2 mAh*/*g after 2000 cycles with a high coulombic efficiency of ≈100% at 5.0 A/g, thus enabling a promising anode material for lithium-ion batteries with high power and energy densities.

## Highlights

• A facile and scalable synthesis of Ta-doped Li_2_ZnTi_3_O_8_ spheres through solid-state reaction using mesoporous anatase TiO_2_ spheres as the self-template
• A combined experimental and theoretical study is conducted to reveal the phase behaviors of Ta-doped Li_2_ZnTi_3_O_8_ during the cyclic charge/discharge process
• Ta^5+^ doping creates rapid electronic transportation channels for high Li^+^ ion diffusion kinetics
• A reversible capacity of 90.2 mAh*/*g after 2000 cycles with a high coulombic efficiency of ≈100% at 5.0 A/g is achieved using optimal Ta-doped Li_2_ZnTi_3_O_8_ spheres

## Graphical abstract

**Figure:**
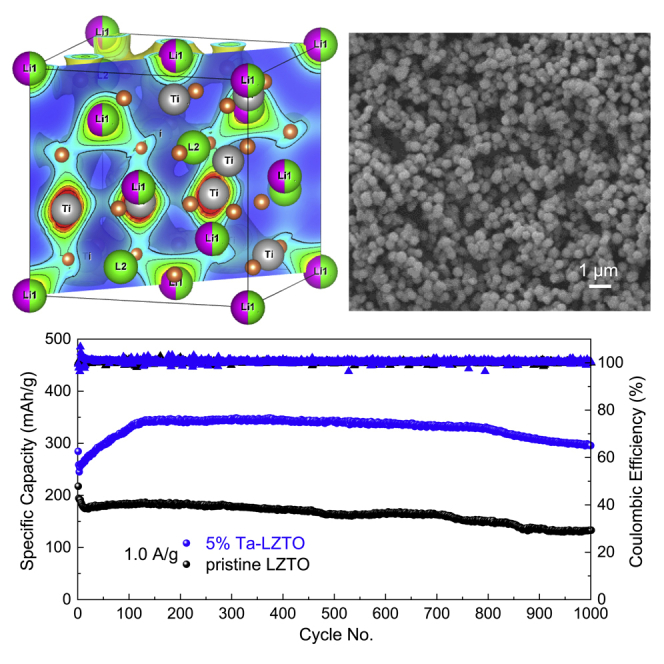

## Introduction

Rechargeable lithium-ion batteries (LIBs) have been widely spread out in portable electronic devices and electric vehicles owing to their integral superiorities in energy density, charge-discharge dynamics, and overall operational lifetime (Tang et al., 2020; Eftekhari, 2019; Yang et al., 2011). Continuous efforts have been devoted to the advanced electrode materials of LIBs to further boost their power density and cycle life and to meet the ever-increasing concerns in the safety. Graphite is the first commercially used low-cost anode material with a lithiation potential below 0.2 V *vs.* Li/Li^+^, which is very close to the lithium stripping voltage (Bai et al., 2019; Feng et al., 2018; Li et al., 2017b). Coupled with its unneglectable volume variation (∼9%), safety issues remain challenging in graphite anode materials. Spinel Li_4_Ti_5_O_12_ with a high redox potential (>1.0 V *vs.* Li/Li^+^) even at high rates has been developed as an alternative anode with almost zero strain in the cyclic process (Kirillov et al., 2018; Wang et al., 2019a; Zhao et al., 2015). However, the relatively low theoretical specific capacity (175 mAh*/*g) restricts its practical applications (Jin et al., 2019).

Cubic spinel Li_2_ZnTi_3_O_8_ (LZTO, space group: *P*4_3_32) possesses an open 3D network in which Li and Ti with a cation ordering of 1:3 locate on the octahedral sites, and Zn occupies the tetrahedral sites, forming a unique (Li_0.5_Zn_0.5_)_tet_[Li_0.5_Ti_1.5_]_oct_O_4_ spatial structure for the reversible intercalation and deintercalation of Li^+^ ions. LZTO shows almost zero volumetric change during the cyclic electrochemical Li^+^ ion insertion/extraction reactions (Chen et al., 2015a; Inamdar et al., 2018), and when compared with LTO, it enables to deliver a higher theoretical specific capacity of 229 mAh*/*g, thereby presenting a promising anode material of LIBs (Hong et al, 2010, 2011). Moreover, because of the lower discharge potential (0.5 V), the LZTO anode can effectively hinder the generation of lithium dendrites and show a better intrinsic safety over graphite (Firdous et al., 2020).

Despite the favorable physicochemical properties, LZTO is still suffering from its poor electrical conductivity and poor high-rate performance because the 3D states of Ti possess wide band gaps (*E*_g_ = 2–3 eV) (Qie and Tang, 2014; Yi et al., 2009). Various modification methods have been developed recently. Surface coating with a conductive carbonaceous layer is a conventional method to enhance the electronic conductivity of LZTO, which, however, frequently sacrifices the volumetric energy density of the resultant LIBs. The fluctuations between rising and falling are also found in MgSiO_3_ (Yang et al., 2019) and La_2_O_3_ (Meng et al., 2019) surface coatings. Alternatively, ion doping into the crystal lattice of LZTO is emerging recently as a facile strategy to enhance the electrical conductivity and the electrochemical performances of doped LZTO anodes (Chen et al., 2018, 2017; Firdous et al., 2020; Li et al., 2017a; Qie and Tang, 2014; Shen et al., 2019; Tang et al., 2014a, 2014b; Chen et al., 2015b; Wang et al., 2019b; Yang et al., 2018; Yi et al., 2015; Zhang et al., 2021, 2020). Unfortunately, the underlying stability of the crystal structure of the ion-doped LZTO during the cyclic charge-discharge process, to our best knowledge, has not been well addressed.

In addition to the chemical modification, developing advanced structures of LZTO on the nanoscale or microscale has been proposed to boost electrochemical energy storage. Solid-state synthesis is the most used method and generally results in irregular bulk structure. The wet-chemical routes, such as sol-gel electrospinning and solution-combustion process have been developed recently (Li et al., 2015a, 2015b; Liu et al., 2016; Tang et al., 2016; Wang et al., 2011; Wu et al., 2019), which, however, involve multiple steps and rely on costly synthesis devices. Moreover, implementing the nanostructured LZTO anodes in LIBs has been developed. The large surface area and small size significantly enhance the initial specific capacity, but the cyclic performance is relatively poor because of the easy distortion of nanostructures.

Strategical combination of sol-gel and hydro/solvothermal processes have been recently developed for the self-template preparation of porous TiO_2_ and ternary perovskite titanate solid and hollow processes. Herein, we propose a novel solid-state process for the synthesis of quaternary Ta-doped LZTO spheres by using mesoporous anatase TiO_2_ spheres (MATS) as the TiO_2_ source and self-template. The spherical morphology of LZTO is well succeeded upon the operation of the Ostwald ripening mechanism during the solid-state reaction. Thus the obtained spherical LZTO (denoted as T0) with an average diameter of 450 nm possesses high structural stability and fast Li^+^ de/intercalation reversibility during the cyclic process. More interestingly, we further demonstrate that the Ta^5+^, having a close ionic radius (0.64 Å) to Ti^4+^ (0.61 Å), is an excellent dopant to substitute Ti^4+^. Upon Ta^5+^ doping by calcination, *d*-block transition Ta^5+^ can result in the defect disorder of LZTO followed by a certain amount of Ti^3+^ generated because of the charge compensation. The charge compensation of Ta^5+^ replacing Ti^4+^ can be realized in the following two ways: 1) the concentration of electrons is increased with the Ta^5+^ doping; 2) the stoichiometric reduction of Ti^4+^ to Ti^3+^ owing to the successful Ta^5+^ doping. As a result, Ta^5+^ doping boosts the electrical conductivity, specific capacity, rate performance, and lifetime of LZTO when employed as the anode material for lithium-ion batteries in both half-cell and full-cell configurations.

## Results

### Solid-state self-template formation and enhanced electrochemical properties of LZTO spheres

**Figure 1:**
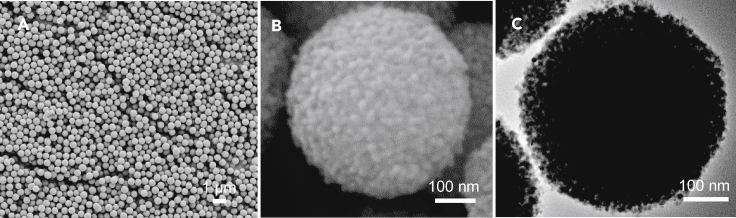
Morphology characterization of MATS (A–C) SEM and (C) TEM image of MATS consisting of anatase TiO_2_ nanocrystallites.

The detailed solid-state self-template synthesis procedure for LZTO spheres is illustrated in Scheme S1. MATS are used as the self-template that are derived from the microwave-induced crystallization of amorphous hydrous TiO_2_ colloidal spheres (HTCS) (Ma et al, 2020, 2021). Compared with conventional hydro/solvothermal process (Ma et al., 2021; Ding et al., 2020), the microwave irradiation can heat up the hydrothermal medium in a prompter and a more homogeneous manner and thus accelerate the *in-situ* crystallization of HTCS with a much shorter time: the hydrothermal reaction time is significantly reduced from the conventional 8‒12 h to 0.5 h. Figures 1A and 1B show MATS synthesized via the microwave-assisted self-templated process; they possess uniform spherical morphology with an average diameter of 450 nm. TEM observation (Figure 1C) verified that MATS consists of ultrafine nanoparticles as the building blocks. XRD analysis shows that MATS are fully crystallized with a single anatase phase (Figure S1A). The grain size, calculated according to Scherrer Equation, is determined to be 18 nm. Moreover, mesopores are generated from the voids among the interconnected anatase nanocrystallites. N_2_ sorption analysis reveals that the surface area of MATS is 219.7 m^2^/g with an average pore diameter of 6.74 nm and a large pore volume of 0.0109 cm^3^/g (Figure S1B).

**Figure 2:**
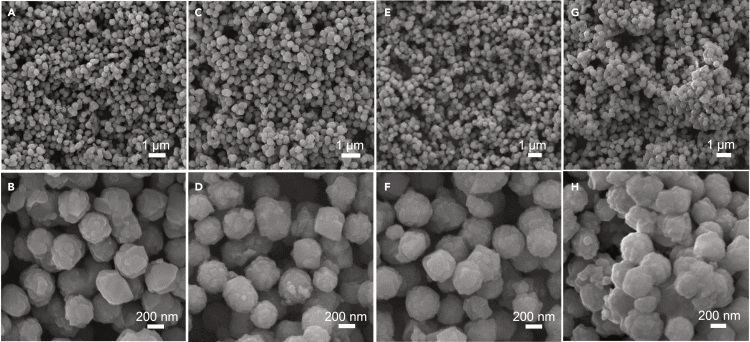
SEM images of the spherical (Ta-doped) LZTO (A–H) SEM images of the spherical (A and B) T0, (C and D) T3, (E and F) T5 and (G and H) T7 derived from the solid-state reaction using MATS as self-template.

The large surface area of MATS with well-defined anatase crystallites and accessible mesopores presents excellent thermal stability and capacity in the solid-state self-template synthesis of LZTO. After mechanically mixing with Li and Zn precursors, followed by mixing with lithium carbonate and zinc acetate and eventually calcined at the optimal 800°C for 4 h, the phase transition from anatase TiO_2_ to LZTO can be readily conducted with excellent preservation of the spherical morphology in the resulting T0 samples, as observed from the SEM images shown in Figures 2A and 2B. The spherical surface consists of densely packed LZTO grains without detectable pores. The BET surface area is calculated to be merely 3.0 m^2^/g (Figure S2A and Table S1).

To understand the solid-state reaction during the formation of LZTO, thermogravimetric-differential thermal analysis (TG-DGA) was conducted. The mixed solid precursors were heated from room temperature to 900°C in the air at a rate of 10°C/min. As shown in Figure S3A, the weight loss in the range from room temperature to 200°C is associated with the evaporation of absorbed H_2_O from the precursor. Then, a sharp weight loss from 200 to 400°C may be related to the decomposition of Zn(Ac)_2_ corresponding to the distinct peak on the DTA curve. Subsequently, in the range of 480–650°C, the weight loss may originate from the decomposition of Li_2_CO_3_. When the temperature exceeds 650°C, a platform appears in the TG curve, which indicates zero weight loss and the successful formation of LZTO phase. We then performed XRD analysis to identify the phase compositions in the samples calcined at different temperatures above 600°C, and found that 800°C for 4h is the optimal sintering condition to obtain the best crystallinity and lowest impurity for T0 (Figure S3B).

The spherical structure of LZTO displays significant monodispersity and packing properties and shows remarkable merits in device fabrication. Compared with LZTO using TiO_2_ nanoparticles (e.g., Aeroxide P25, Hombikat 8602 and rutile TiO_2_) as the TiO_2_ sources, our LZTO spheres with higher phase purity and less aggregation show dramatically enhanced specific capacities, rate, and cyclic performances in the LIB applications (Figures S2−S7).

### Phase composition and textural properties of Ta-doped LZTO spheres

Unlike the conventional self-template synthesis of titanate spheres by hydro/solvothermal process (Pan et al., 2015; Zhang and Zhang, 2019), the currently developed solid-state self-template method without using the critical condition and liquid phase greatly eases the mass production. Moreover, it allows obtaining thermodynamically stable phases through solid-state diffusion, exhibiting great advantages in the modification of LZTO spheres. Ta^5+^ ions can be further doped into LZTO crystal lattices by simply introducing Ta precursor into the ball milling powder, and various Ta-doped LZTO (Li_2_ZnTi_3-*x*_Ta_*x*_O_8_) spheres can be obtained upon calcination. Herein, with synthetic chemical inputs *x* = 0, 0.03, 0.05, 0.07, and so on, we denote the pure and Ta-doped LZTO as T0, T3, T5, and T7. Similar to T0, the spherical morphologies of the resultant T3, T5 and T7, as shown in Figures 2C–2H, are also well inherited from MATS after the solid-state reaction between MATS and the foreign Li, Zn, and Ta species at high calcination temperatures. Besides, the specific surface area of T0 was not significantly affected upon doping with different amounts of Ta (Figure S2B and Table S1). Representative EDS mapping images for T5 (Figure S8) further confirm that the homogeneous distributions of Zn, Ti, O, and Ta.

**Figure 3:**
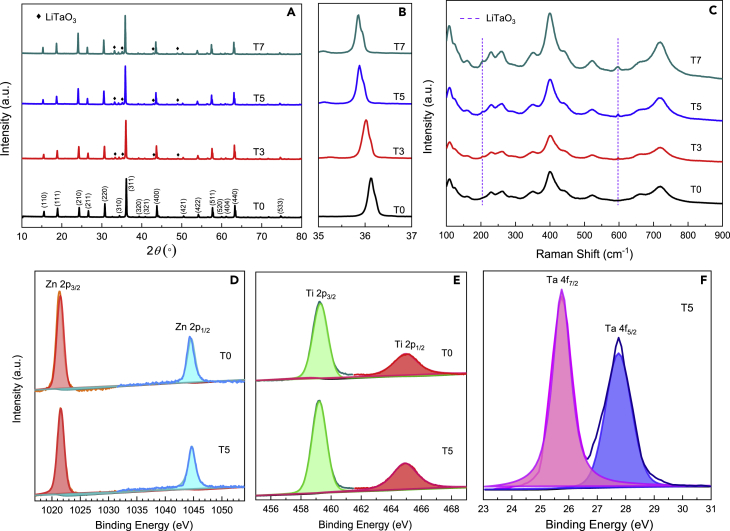
XRD, Raman, and XPS measurements of (Ta-doped) LZTO spheres (A–F) Phase and composition analyses of T0, T3, T5, and T7: (A) XRD profiles; (B) Partially magnified (311) diffraction peak at 2*θ* = 35–37°; (C) Raman spectra; and high-resolution XPS spectra of Zn (D), Ti (E), and Ta (F) in T0 and T5.

Figure 3A shows the XRD profiles of Ta-doped LZTO samples, which can be indexed as the cubic spinel LZTO with *P*4_3_32 (International Centre for Diffraction Data [ICDD] No. 44-1037). T0 shows high phase purity without any additional phase. Upon doping with Ta^5+^ ions, a gradual shift to the lower angle in the diffraction peaks was observed, as can be seen from (311) peak in Figure 3B, thus suggesting the expansion in the volume of crystal lattices owing to the larger size of Ta^5+^ over Ti^4+^ that are doped into the crystal lattice of LZTO. Meanwhile, a new crystal phase of LiTaO_3_ (ICDD No. 26-1190) appears, and its diffraction peaks gradually intensify with the increase in the dopant.

To precisely confirm crystal parameter and phase composition, we refined the XRD patterns with the TOPAS (TOtal PAttern Solution) program (Kartini et al., 2018). The Rietveld refinement results (Figure S9A–S9D) are acceptable based on the reasonable evaluation parameters of *R*_*p*_ and *R*_*wp*_. Table S2 shows that the substitution of Ti^4+^ with larger sized Ta^5+^ truly expands the lattice parameters of LZTO, which might broaden the transport channels of Li^+^ and the resultant electrochemical properties of the LZTO anode. According to our refinements, 1.25, 3.50, 4.17 wt% of LiTaO_3_ exists in T3, T5, and T7 samples, corresponding to 2.49, 3.52, 5.23 wt% Ti^4+^ ions that are substituted by Ta^5+^, respectively. Thus, over 70% Ta^5+^ ions are successfully doped into the LZTO lattice structure for all samples. Merely, a limited amount of Ta converts to the piezoelectric LiTaO_3_ phase although its amount is gradually increased with the increase in *x*.

The presence of LiTaO_3_ in Ta-doped LZTO samples was also confirmed by Raman spectra. As shown in Figure 3C, all four samples show clear bands at around 106, 400, and 719 cm^−1^ that can be attributed to LZTO. With Ta doping, three additional Raman peaks at 150, 200, and 600 cm^−1^ appear, which can be indexed to LiTaO_3_. With the increase in synthetic chemical input of Ta dopant, the Raman vibrations from LiTaO_3_ strengthen gradually. XPS survey spectra in Figure S10 show the existence of Li, Zn, Ti, O, and Ta elements on the surface. Figures 3D–3F show the high-resolution XPS spectrum of Zn^2+^ 2p_3/2_ (1021.5 eV), Zn^2+^ 2p_1/2_ (1044.6 eV); Ti^4+^ 2p_3/2_ (459.3 eV), Ti^4+^ 2p_1/2_ (456.1 eV) and Ta^5+^ 4f_7/2_ (25.75 eV), Ta^5+^ 4f_5/2_ (27.55 eV) indicating the existence of fully oxidized Zn^2+^, Ti^4+^ and Ta^5+^ species in T5.

The TEM image of T5 (Figure S11A) shows the monodispersed spherical particles with sizes in the range of 420‒450 nm. Besides, a crystallin LiTaO_3_ layer on the surface of T5 sample particles can be distinctly observed according to the high-resolution TEM resolution. As shown in Figure S11B, the lattice fringes with the *d*-spacing of ∼4.80 Å is corresponding to the (111) plane of LZTO, and a *d*-spacing of about 3.72 Å in the surface region is the typical (012) crystal plane of LiTaO_3_.

### Density-functional theory (DFT) calculations and electron density distributions of Ta-doped LZTO spheres

**Figure 4:**
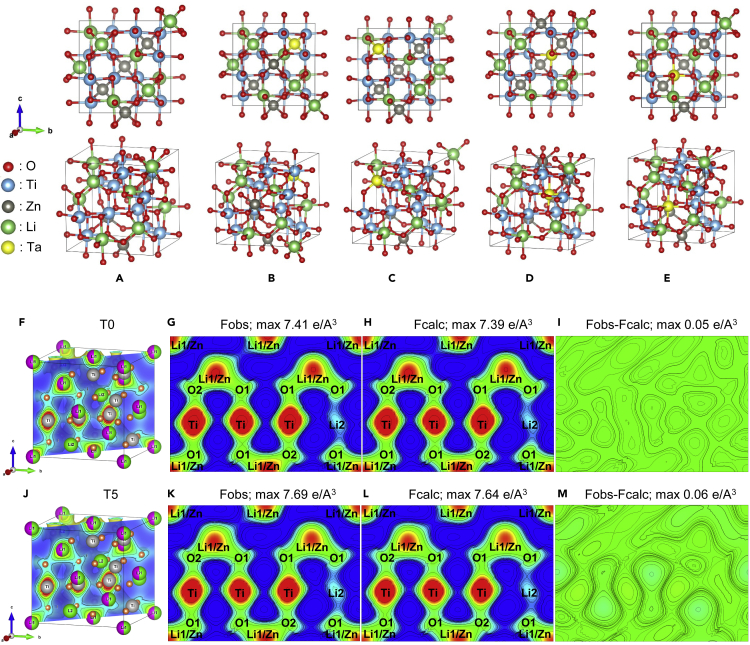
Crystal structures of LZTO and Ta-doped LZTO through four probable substitutions; 3D crystal structures and electron density maps of T0 and T5 (A–M) The crystal structures of (A) LZTO and Ta-doped LZTO through four probable substitutions: Zn↔Ta (B), tetrahedral Li↔Ta (C), octahedral Li↔Ta (D), and Ti↔Ta (E); 3D crystal structure (F, J) and electron density maps (G–I) of T0 and (K–M) T5, respectively.

DFT calculations have been conducted to confirm the exact location of Ta^5+^ dopants in the crystal lattices of LZTO. Two possible substitution sites are available for the guest Ta atoms: tetrahedral sites (Li or Zn atoms) or octahedral sites (Li or Ti atoms). Correspondingly, there are four substitution possibilities: Li (tetrahedral site)↔Ta, Zn (tetrahedral site)↔Ta, Li (octahedral site)↔Ta, and Ti (octahedral site)↔Ta, as shown in Figure 4. Their calculated formation energies, as summarized in Table 1, are determined to be −34.82, −35.43, −34.82, and −35.72 eV, respectively. Note that the formation energy for standard LZTO is calculated to be −35.18 eV per formula. Thus, the Ti↔Ta substitution is the most favorable because of the lowest formation energy required.

**Table 1:** The calculated formation energy (*E*_f_), lattice, and volume of Ta-substituted LZTO

	Standard	Tetrahedral site substitution		Octahedral site substitution	
		Zn↔Ta	Li↔Ta	Li↔Ta	Ti↔Ta
Unit cell	Li_8_Zn_4_Ti_12_O_32_	Li_8_Zn_3_Ti_12_TaO_32_	Li_7_Zn_4_Ti_12_TaO_32_	Li_7_Zn_4_Ti_12_TaO_32_	Li_8_Zn_4_Ti_11_TaO_32_
Lattice (Å)	8.38	8.41	8.42	8.41	8.41
Volume (Å^3^)	588.05	594.66	595.49	597.23	591.80
*E*_f_ (eV)/formula	−35.18	−35.43	−34.82	−34.82	−35.72

Attentions have been also paid to the volume expansion upon the Ti↔Ta substitution. The calculated lattice parameters fall in the range of 8.38–8.42 Å, which is in good agreement with our XRD results (8.37 Å for standard LZTO and 8.38 Å for Ta-LZTO) (Yang et al., 2017). In addition, the calculated unit cell volume of the standard LZTO is 588.05 Å^3^, which is slightly increased to 591.80 Å^3^ after the Ta substitution.

The electron density distributions of the system were calculated by the maximum entropy method. The structure factors obtained from Rietveld refinements of XRD patterns in Figure 3A were reconstructed and visualized with Visualization for Electronic and STructural Analysis programme. Figures 4F and 4J show the 3D unit cells of T0 and T5 with (110) lattice plane shaded with iso-surfaces inside the unit cells, respectively. Figures 4G, 4H, 4K, and 4L show their 2D charge density distribution maps with contour on the (110) lattice plane. Herein, Zn (*z* = 30) dominates over Li1 (*z* = 3), Wyckoff 8c because of its higher number of electrons, but its density distribution is lower than that of Ti (*z* = 22), Wyckoff 12d, because of the partial occupation with Li. The O atom and its covalent bond can also be seen from both maps as well as the Li2 because of the high quality of data. The presence of Ta (*z* = 73) dopant substituting Ti lattice can be seen by comparing the normalized Ti density distribution. The density distribution of Li1/Zn and Li2 decreases as the Ta substitution takes place.

The difference in Fourie map (Fobs-Fcalc) is an indication between the observed data and modeled structure. Figure 4I shows the highest peak on the pure system of T0 is 0.09 e/A^3^, while Figure 4M where the structure is refined with the substitution of Ta in partial Ti lattice in T5 shows the highest peak of 0.11 e/A^3^. Both show an exceptionally low electron density as shown by almost monotonous color distribution. These results agree with the DFT calculation, proving the successful substitution of Ta^5+^ with Ti^4+^ in LZTO lattices.

### Charge/discharge mechanism of Ta-doped LZTO spheres

To reveal the electrochemical reaction mechanism of T0 and T5, their half-cells were subjected to the *in-situ* XRD analysis. The reversible insertion/extraction reaction of the LZTO can be written as the following Equation 1:

(Equation 1) Li_2_ZnTi_3−*x*_Ta_*x*_O_8_ + 3Li^+^ + 3e^−^ → Li_5_ZnTi_3−*x*_Ta_*x*_O_8_

**Figure 5:**
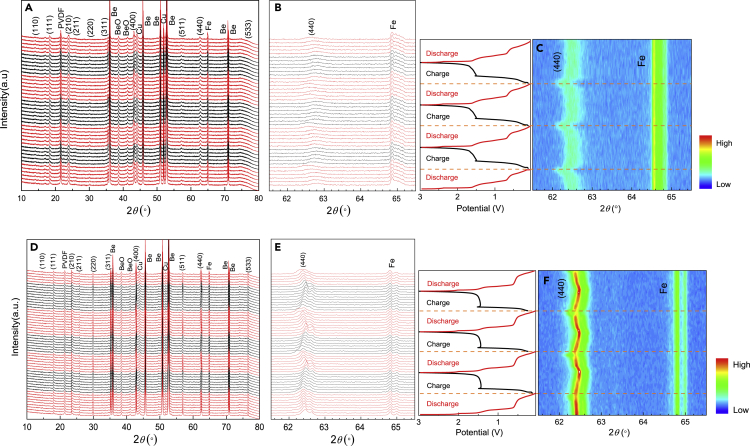
*In-situ* XRD patterns, and 2D contour plots of T0 and T5 anodes (A–F) *In-situ* XRD patterns of (A) T0 and (D) T5 anodes after first discharge process and upon three charge/discharge cycles; (B and E) Corresponding magnified (440) peak profiles; (C and F) 2D contour plots with the corresponding galvanostatic charge/discharge curves of T0 and T5 anodes, respectively.

As shown in Figures 5A and 5C, the crystal structure of spinel LZTO in T0 and T5 was not destroyed without the generation of new crystal phases at the first three cycles of the charge-discharge process after ignoring the diffraction peaks from the *in-situ* test molds, PVDF, and Cu, suggesting the high reversibility of Equation 1 operating in T0 and T5 anodes.

Figures 5B and 5E show the amplified patterns in the 2*θ* = 61.5–65.5°. During the cyclic charging/discharging process, the (440) peak slightly shifts toward higher/lower angles, accompanied by the reversible de/intercalation of Li^+^. Figures 5C and 5F compare the corresponding 2D contour plots that originated from the (440) diffraction peaks from the *in-situ* XRD patterns. Compared with T0, T5 shows less significance in peak shifts during the first three cycles. The superiority in the structural stability of T5 is mainly ascribed to their larger unit cell volume with higher tolerances in structural expansion and contraction during the cyclic lithiation and delithiation processes.

**Figure 6:**
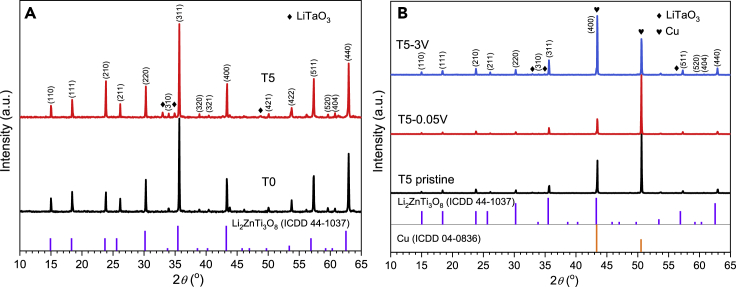
Synchrotron high-energy XRD patterns of T0 and T5 (A and B) Synchrotron high-energy XRD patterns of powered T0 and T5 spheres; (B) *Ex-situ* synchrotron high-energy XRD patterns of T5 anode at different potential states after 100 cycles of charge/discharge process.

To better understand the underlying mechanism of the cycling properties upon Ta^5+^ doping, the synchrotron high-energy XRD technique was employed to investigate the crystal variations in T0 and T5 anodes after cyclic charge/discharge processes. To confirm the peak position, we measured the powered T0 and T5 firstly. As shown in Figure 6A, the diffraction peaks of LiTaO_3_ can be readily resolved in T5 and with no impurity in T0. We then measured the phase composition of T0 and T5 anodes at the pristine state and upon charge/discharge process, specifically discharged at 0.05 V and charged to 3.0 V after 100 cycles. Figure 6B shows the *ex-situ* synchrotron high-energy XRD patterns of T5 anode with Cu substrates. Note that there is an overlapping between LZTO (400) and Cu (111) at 2*θ* of 43.4°. LZTO and LiTaO_3_ can be clearly observed in T5 at two different voltage states. No additional phases generate upon the electrochemical lithium storage reactions, and the intensities of the diffraction peaks for spinel LZTO phases are increased with the increase in voltage, indicating the significant structural stability of LZTO for cyclic performances. However, the peak position and intensity of LiTaO_3_ remain almost unchanged, suggesting its inertness in lithium storage. Thus, LiTaO_3_ is assumed not to participate in the insertion/extraction reactions of Li^+^ ions.

### Electrochemical properties of Ta-doped LZTO spheres

The initial charge/discharge curves of T0, T3, T5, and T7 samples tested at 0.1, 0.5, and 5 A/g are depicted in Figure S12A–C, respectively. All Ta-doped LZTO show a higher specific discharge capacity and a higher charging and discharging platform than T0. This trend becomes more obvious when the measurement is conducted at the high current density. For instance, at a high current density of 5A/g, the charge/discharge capacities of T0, T3, T5, and T7 in the first cycle are 199.2/202.7, 218.4/218.8, 230.2/225.6, 213.9/215.7 mAh*/*g, corresponding to the coulomb efficiencies of 98.3%, 99.8%, 102.0%, and 99.2%, respectively. The Ta^5+^ doping has a great influence on the electrochemical performances of LIBs. Even at a small amount of 3%, a significant improvement in LIB properties has been achieved. The optimum doping concentration of Ta^5+^ ions for LZTO is determined to be 5%, at which T5 delivers the best cyclic performance and the highest specific capacity. Our XRD analysis and refinement have demonstrated that the amount of Ta^5+^ dopants increase gradually with the increase in value of *x*, accompanied by the enrichment in LiTaO_3_ species coating on the surface synchronously. According to the recent studies on piezoelectric materials modified LIB electrodes (Lee et al., 2016; Si et al., 2020), piezoelectric oxides such as BaTiO_3_ and LiTaO_3_ enable generating an internal piezoelectric field to promote Li^+^ diffusion. Typically, with the LiTaO_3_ layer, the insertion/extraction of Li^+^ ions in LZTO will induce the crystal stress on the piezoelectric surface coating, creating a local electric field at the electrode-electrolyte interphase and thus guiding fast Li^+^ diffusion kinetics. However, LiTaO_3_ itself is insulating and unable to store Li^+^ ions. Therefore, the excessive LiTaO_3_ (i.e., *x* = 7%) might greatly retard the charge/discharge process.

**Figure 7:**
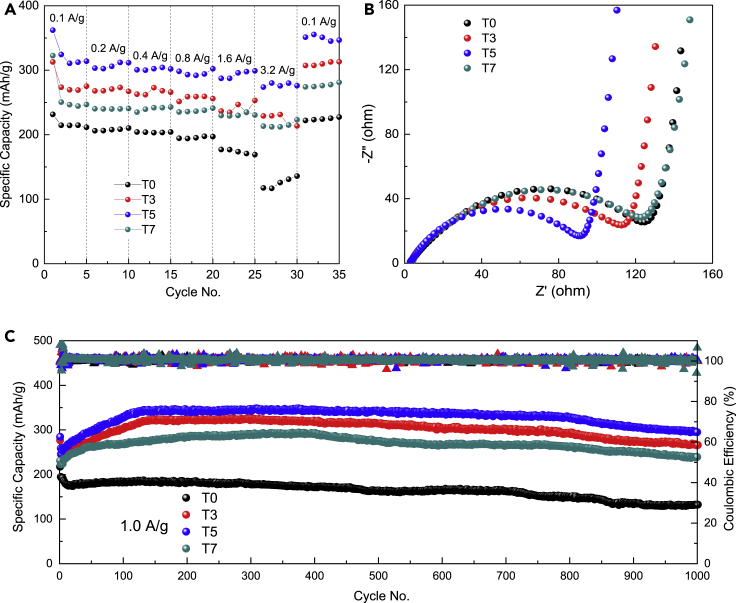
Electrochemical performances of spherical (Ta-doped) LZTO anodes in half cells (A–C) Electrochemical performances of T0, T3, T5 and T7 anodes in half cells: (A) rate performances at different current densities of 0.1–3.2 A/g in 0.05–3.0 V (*vs.* Li^+^/Li); (B) open-circuit EIS spectra; and (C) cycling performances and Coulombic efficiencies at 1.0 A/g.

Figure 7A shows the rate performances of T0, T3, T5, and T7 in the voltage range of 0.05–3.0 V (*vs.* Li/Li^+^) at 0.1–3.2 A/g. The capacity is dramatically increased with the Ta^5+^ doping. The anodes can be activated after the preliminary cycling. Typically, the optimal T5 shows the highest coulomb efficiency at all charge-discharge rates. The highest initial specific capacities of 362.1 mAh*/*g at 0.1 A/g can be delivered by T5, and even at 3.2 A/g, an impressive discharge capacity of 273.9 mAh*/*g can be retained. Therefore, T5 exhibits the best rate performances.

Figure 7B shows their corresponding open-circuit EIS spectra. All anodes exhibit similar Nyquist plots comprised of a semicircle at the high-frequency region and a straight line at the low-frequency region. The diameter of the semicircle is significantly reduced upon Ta^5+^ doping, suggesting that Ta^5+^ doping and LiTaO_3_ coating can effectively reduce the charge transfer resistance. T5 has a minimum impedance of 92.5 Ω, which is helpful to improve the Li^+^ de/intercalation kinetics.

Long-term cycling stabilities of T0 and Ta-doped samples tested at different current densities of 0.5, 1.0, and 5.0 A/g are summarized in Figures 7C and S13. All Ta-doped samples deliver larger specific capacities than T0 during the entire cycling process. At *x* = 0.03, a significant improvement has been realized, implying that Ta^5+^ doping plays a key role in the electrochemical performance enhancement, because merely a track amount of LiTaO_3_ (1.21 wt%) exists in the T3 sample. After 1000 cycles, T5 shows 317.2 and 294.9 mAh/g with coulomb efficiencies of ≈100% at 0.5 and 1.0 A/g, respectively. Impressively, at an ultrahigh current density of 5.0 A/g, T5 delivers an initial discharge capacity of 235.8 mAh*/*g and remains at 90.2 mAh*/*g after 2000 cycles. To the best of our knowledge, the rate capability and the cycling performances of the T5 sample are one of the best results among various LZTO-based LIB anode materials (see Table S3).

Attempts have been further made to study the electrochemical properties of La-doped LZTO spheres in the full battery by using commercial LiFePO_4_ as the cathode. Figure S14 compares the lithium storage performances of T5/LiFePO_4_ and T0/LiFePO_4_ in full cells. Ta^5+^ doping significantly improves the electrochemical performances of LZTO. Impressively, T5/LiFePO_4_ possesses a higher and more stable discharge voltage platform (≈1.98 V) than T0/LiFePO_4_ (≈1.85 V) (Figure S14A). Moreover, Ta^5+^ doping shows significant improvement in the capacity performances again: The initial discharge capacity and coulombic efficiency at 0.5 A/g are (154.7 mAh/g, 92.5%) and (148.7 mAh/g, 90.2%) for T5/LiFePO_4_ and T0/LiFePO_4_ full cells. After 200 cycles, the capacities decrease to 119.3 mAh/g and 99.7 mAh/g with retentions of 77.1% and 67.0% (Figure S14B), respectively. The excellent electrochemical properties of optimal T5 operated in both half and full cells are mainly because of Ta^5+^ doping with large unit cell volume, high stability in the crystal structure, appropriate amount of LiTaO_3_ surface coating, monodispersity with less aggregation, spherical morphology with good packing properties, low impedance, and high Li^+^ transportation kinetics.

### Effect of Ta^5+^ doping on the electrochemical kinetics during the cyclic process

**Figure 8:**
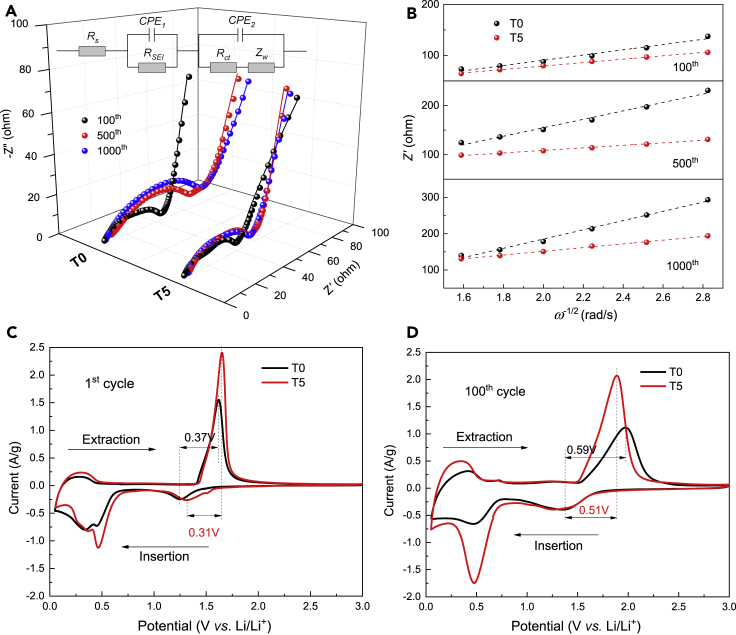
Nyquistplots and CV characterization of T0 and T5 (A–D) (A) Nyquist plots and the fitted results (solid line) with the equivalent circuit (inset); (B) the relationship curves between *Z′* and *ω*^−1/2^ in the low-frequency region of T0 and T5 at their 1^st^, 500^th,^ and 1000^th^ cycles at 25°C; their CV plots in the voltage range of 0.05–3.0 V at (C) 1^st^ and (D) 100^th^ cycles at a scanning rate of 0.5 mV/s.

To gain a deeper insight into the effect of Ta^5+^ doping on the performances, electrochemical impedance spectroscopy (EIS) and cyclic voltammetry (CV) analyses have been conducted to unveil the electrochemical kinetics and polarization at different cycles. Figure 8A compares the EIS spectra obtained from the half cells at the 100^th^, 500^th^, and 1000^th^ cycles at 5A/g with a charge potential of 1.5 V. The Nyquist plots are composed of two semicircles and a sloped tail, and the fitting result is simulated based on the equivalent circuit including the electrolyte resistance (*R*_s_), SEI film resistance (*R*_SEI_), charge transfer resistance (*R*_ct_), constant phase elements (*CPE*_1_*, CPE*_2_) and Warburg impedance (*Z*_w_) that are directly related to Li^+^ diffusion resistance (Wang et al., 2016; Yang et al., 2015). The fitting results of *R*_s_, *R*_SEI_, *R*_ct,_ and Li^+^ ion diffusion coefficient (*D*_Li+_) are listed in Table 2. The total resistance (*R*_T_ = *R*_s_ + *R*_SEI_ + *R*_ct_) of T5 is much less than that of T0 at 100^th^, 500^th^, or 1000^th^ cycles, respectively, indicating the enhanced electronic conductivity because of the Ta^5+^ doping.

**Table 2:** EIS fitting results for T0 and T5

Sample	*R*_s_/Ω			*R*_SEI_/Ω			*R*_ct_/Ω			*σ*_w_/cm^2^ ·s^−1/2^			*D*_Li+_/×10 ^−13^ cm^2^ ·s^−1^		
	100^th^	500^th^	1000^th^	100^th^	500^th^	1000^th^	100^th^	500^th^	1000^th^	100^th^	500^th^	1000^th^	100^th^	500^th^	1000^th^
T0	5.0	6.9	5.9	38.1	60.24	65.3	4.9	10.58	25.2	51.5	85.9	127.2	0.36	0.13	0.06
T5	3.7	6.6	4.7	25.4	44.3	64.8	3.8	3.2	10.2	33.5	25.8	51.2	0.86	1.46	0.37

The real part of the Nyquist plot can be expressed using Equation 2:

(Equation 2) $$Z^{\prime }=R_{\text{s}}+R_{\text{ct}}+\sigma_{\text{w}}\omega^{-1/2}$$

where *ω* (2π*ƒ*) is the angular frequency in the low-frequency region. *σ*_w_ is the Warburg factor related to mass transport. Figure 8B shows the relationship between *Z′* and *ω*^*−*1/2^ then the *σ*_w_ can be obtained from the slope. The *D*_Li+_ values can be calculated according to Equation 3:

(Equation 3) $$D_{\text{Li}+}=R^{2}T^{2}/\left(2A^{2}n^{4}F^{4}C^{2}\sigma_{\text{w}}^{2}\right)$$

where *R* = 8.314 J/mol·K, *T* = 298.5 K, *n* = 1, *F* = 96,485 C*/*mol, *C* = 0.01698 mol*/*cm^3^, and *A* = 1.13 cm^2^ in this work. The *D*_Li+_ of the T0 sample decreases gradually with the increase in the number of cycles; however, the *D*_Li+_ of the T5 sample increases at first and then decreases. This phenomenon indicates that the ohmic polarization for lithiation/delithiation has been effectively inhibited after Ta^5+^ was induced. Besides, the values of *D*_Li+_ for T5 are higher than that for T0 after the 100^th^, 500^th^, and 1000^th^ cycles (Table 2). The above results prove that the successful Ta^5+^ doping is beneficial to accelerate the Li^+^ diffusion coefficient, leading to dramatic enhancements in the rate and cyclic performance of T5.

CV measurements were also performed at the different cycles (1^st^, 100^th^, 200^th^, and 300^th^) at a potential scan rate of 0.5 mV/s between 0.05 and 3.0 V (*vs.* Li^+^/Li) to further compare the electrochemical properties of T0 and T5. The results are shown in Figures 8C, 8D, and S15A and S15B, respectively. The redox reaction process greatly differs between the 1^st^ and the subsequent cycles for T0 and T5. The potential difference (*ϕ*_p_) between the cathodic and anodic peaks can reflect the degree of polarization. In the 1^st^ cycle, the *ϕ*_p_ is 0.37 and 0.31 V for T0 and T5 respectively, and the gap becomes larger gradually with the proceeding of cyclic charge/discharge process. At the 100^th^, 200^th^, and 300^th^ cycles, the values are (0.59, 0.51) (0.81, 0.75) (1.00, 0.80), respectively. Apparently, T5 keeps a lower *ϕ*_p_ value than that for T0 during the cyclic process, which manifests the weakened polarization in T5 and better electrochemical kinetics, and well matches with the electrochemical performances and EIS results as shown in Figures 7A–7C and 8A and 8B, respectively.

Attempts have been made to obtain insights into the Li^+^ diffusion kinetics during the rate performances for different LZTO samples. As shown in Figure S16, their CV analyses were carried out at various scan rates (0.1, 0.2, 0.4 and 0.8 mV/s) between 0.05 and 3.0 V (*vs.* Li^+^/Li), among which T5 possesses the largest redox area. The relationship between peak current density (*i*_p_) of cathodic/anodic reaction and the square root of scan speed (*v*^0.5^) can be also plotted, and the *D*_Li_^+^ of all samples can be calculated using the Randles-Sevcik Equation:

(Equation 4) $$i_{\text{p}}=2.69\times 10^{5}\times n^{1.5}SCD_{{\mathrm{Li}}^{+}}^{0.5}\nu^{0.5}$$

where *n*, *S,* and *C* refer to the charge transfer number, the surface area of LZTO per unit weight, and the molar concentration of Li^+^ ions in solid, respectively. The *D*_Li+_ values for T5 are 2.66 × 10^−13^ (delithiation), 0.29 × 10^−13^ (lithiation), and 1.76 × 10^−13^ (lithiation) cm^2^/s, which are better than 1.83 × 10^−13^ (delithiation), 0.09 × 10^−13^ (lithiation), and 0.53 × 10^−13^ (lithiation) cm^2^/s for T0, respectively.

Moreover, the chemical diffusion coefficient of Li^+^ in the LZTO anode materials was conducted using galvanostatic intermittent titration technique (GITT) to better understand the difference in electrochemical performances upon Ta^5+^ doping. Figure S17 shows the chemical diffusion coefficients of Li^+^ in T0 and T5 as a function of voltage for the 100^th^ cycle with a constant current density of 100 mA/g and each pulse lasts 300 s followed by 600 s of rest. As shown from Figure S17A and S17B, T5 takes a longer time for Li^+^ to reach a steady-state and possesses a larger capacity than T0.

The GITT diffusivity of our LZTO anode materials can be expressed as:

(Equation 5) $$D_{{\mathrm{Li}}^{+}}=\frac{4}{\pi \tau }{\left(\frac{m_{B}V_{m}}{M_{B}A}\right)}^{2}{\left(\frac{\Delta V_{s}}{\Delta V_{t}}\right)}^{2}\;\left(\tau \ll L^{2}/D_{{\text{Li}}^{+}}\right)$$

where *τ* is the time duration of the pulse; *m*_B_, *V*_m_,and *M*_B_ are the active mass, molar volume, and molecular weight of LZTO, respectively; *A* is the cell interfacial area; *L* is the thickness of the electrode; Δ*V*_s_ means the change in the steady-state voltage during the respective single titration; and Δ*V*_t_ is the total transient change in cell voltage after subtracting the IR drop (Shen et al., 2013).

As calculated from Figure S17C and S17D, the *D*_Li+_ values of T5 during the 100^th^ charge/discharge process are determined to 10^−9.0^ to 10^−11.5^ cm^2^/s, which are higher than those of T0 (10^−10.0^ to 10^−12.0^ cm^2^/s). Therefore, the *D*_Li+_ values at the charge and discharge states are closer for T5, further suggesting its higher performances in the reversible electrochemical insertion/extraction of Li^+^ ions.

## Discussion

A self-template solid-state synthesis has been developed to prepare Ta-doped LZTO (Li_2_ZnTi_3-*x*_Ta_*x*_O_8_) spheres as a durable anode material of LIBs. Our theoretical DFT calculation and XRD analysis confirm that the Ta^5+^ ions can be uniformly doped into crystal lattices of LZTO through the Ti↔Ta substitution. The Ta^5+^ doping enhances the electronic conductivity and expands the crystal volume of LZTO, and helps to create rapid electronic transportation channels for the rapid Li^+^ ion diffusion kinetics. In addition, a small amount of piezoelectric LiTaO_3_ exists as the secondary phase on the spherical surface, which improves the electrical contact and interfacial Li^+^ transport kinetics. The elaborated Ta-doped LZTO spheres exhibit superior Li^+^ storage capability and outstanding structure stability during the cycling process. Typically, at the optimale synthetic chemical input (*x* = 0.05), Li_2_ZnTi_3-*x*_Ta_*x*_O_8_ anodes with 3.50 wt% LiTaO_3_ coating exhibit an initial capacity of 230.2 mAh/g at 5 A/g and a reversible capacity of 90.2 mAh*/*g after 2000 cycles with a high coulombic efficiency of ≈100%, thus demonstrating a promising LIB anode material.

### Limitations of the study

The Ta^5+^ doping enables a durable and high-capacity LIB anode material but seems unable to sufficiently decrease the redox potentials of Ta-doped LZTO spheres when working in half-cells and full-cells. Our future efforts will be devoted to addressing this issue.

## STAR★Methods

### Key resources table

**Table:** 

REAGENT or RESOURCE	SOURCE	IDENTIFIER
**Chemicals, peptides, and recombinant proteins**		

titanium (IV) tetraisopropoxide (>99%)	Sigma-Aldrich	CAS#546-68-9
Ethanol (≥99.7%, HPLC)	Sigma-Aldrich	CAS#64-17-5
Ammonium hydroxide solution (25-28%, GR)	Sigma-Aldrich	CAS#1336-21-6
Anhydrous Acetonitrile (99.99%, HPLC)	Sigma-Aldrich	CAS#75-05-8
Lithium carbonate (99.99%, metals basis)	Sigma-Aldrich	CAS#554-13-2
Tantalum oxide (99.99%, metals basis)	Sigma-Aldrich	CAS#1314-61-0
Zinc acetate (99.99%, metals basis)	Sigma-Aldrich	CAS#557-34-6
Aeroxide P25	Sigma-Aldrich	CAS#13463-67-7
Hombikat 8602 (∼100nm)	Sigma-Aldrich	CAS#1317-80-2
Rutile TiO_2_ (∼50 nm)	Sigma-Aldrich	CAS#13463-67-7

**Critical commercial assays**		

Neware 5V10mA battery tester	Neware, China	https://www.neware.com.cn/
LIB-XRD-03C battery case	Zhongke Wanyuan Technology	http://www.zkwy888.com/

**Deposited data**		

PDF-4+ 2021	ICDD	https://www.icdd.com/

**Software and algorithms**		

ZIVE MP1 electrochemical workstation	WonATech Corp., Korea	http://www.wonatech.com/
CHI760E electrochemical workstation	CH Instruments	http://www.chinstr.com/
Neware battery testing system	Neware, China	https://www.neware.com.cn/
VESTA v3.5.7	K. Momma & F. Izumi	http://jp-minerals.org/vesta/en/
TOPAS v6	Bruker	https://www.bruker.com/
Origin 2020	Originlab	https://www.originlab.com/

**Other**		

Powder X-ray diffraction	Rigaku SmartLab SE, Japan	https://japan.rigaku.com/
Grazing incidence X-ray diffraction	Beijing Synchrotron Radiation Facility	http://english.bsrf.ihep.cas.cn/
Scanning Electron Microscopy	JSM-7800F, Japan	https://www.jeol.co.jp/
Transmission Electron Microscopy	JEM-2010, Japan	https://www.jeol.co.jp/
Oxford X-MAX50 energy dispersive spectrometer	Oxford Instruments, UK	https://www.oxinst.com/
XPS spectra	Thermo Fisher Scientific Inc., USA	https://www.thermofisher.com/
Raman spectra	Horiba Lab RAM HR	https://www.horiba.com/
Brunauer-Emmett-Teller	Micromeritics, ASAP 2020 HD88	https://www.micromeritics.com/
Thermogravimetry	NETZSCH, STA 2500, Germany	https://www.netzsch-thermal-analysis.com/

### Resource availability

#### Lead contact

Further information and requests for resources should be directed to and will be fulfilled by the lead contact: Jia Hong Pan (pan@ncepu.edu.cn)

#### Materials availability

This study did not generate new unique reagents.

### Method details

#### Preparation of MATS by the microwave-assisted self-template route

Scheme S1 shows the diagram of our synthesis of T0. MATS was derived from microwave hydrothermal crystallization of HTCS (Ma et al., 2021, Pan et al., 2014). Briefly, TTIP (4.0 ml) was precipitated in a mixed solution containing ethanol (19.2 g), acetonitrile (12.8 g), DI water (0.15 g) and diluted NH_3_·H_2_O (0.068 ml, ∼28%) under vigorous stirring. After stirring for 5 min, the suspension was aged for 3 h under gentle stirring at room temperature. After centrifugation and washing, the as-synthesized HTCS were redispersed in DI water and subjected to microwave irradiation (Power: 150 W; temperature: 130°C) for 0.5 h by using CEM Discover SP equipment under continuous stirring.

#### Optimization of LZTO spheres by solid-state self-template route

MATS, Li_2_CO_3_ and zinc acetate with a stoichiometric amount were ball-milled at 150 rpm for 4 h in the 100 ml vessel of the ball-milling jar with ethanol as the dispersant. The mixed powders were finally calcined at the optimal 800°C for 4 h in air. The resulting spherical LZTO sample was denoted as T0.

To investigate the effect of TiO_2_ sources on the final electrochemical performances, other LZTO samples were synthesized under similar conditions were prepared using commercial TiO_2_ including Aeroxide P25, Hombikat 8602, and Rutile TiO_2_ (Aldrich, ∼50 nm), and the resultant samples were denoted as P25-LZTO, 8602-LZTO, and R-LZTO respectively.

#### Preparation of Ta-doped LZTO spheres

Li_2_ZnTi_3-*x*_Ta_*x*_O_8_ (synthetic chemical input *x* = 0.03, 0.05, 0.07) spheres were prepared via a similar procedure for T0. MATS, Li_2_CO_3_, zinc acetate, and Ta_2_O_5_ with a stoichiometric amount were ball-milled at 150 rpm for 4 h in the 100 ml vessel of the ball-milling jar with ethanol as the dispersant. The mixed powders were finally calcined at 800°C for 4 h in air. The obtained samples with different Ta^5+^ dopants (*x* = 0.03, 0.05, 0.07) were marked as T3, T5, T7, respectively.

#### DFT calculation methodology

DFT calculations were performed by using the plane-wave technique implemented in Vienna ab initio simulation package (Kresse and Hafner, 1993a, Kresse and Hafner, 1993b). The generalized gradient approximation with the Perdew-Burke-Ernzerhof (PBE) functional has been employed to describe the exchange-correction potential in all calculations (Blöchl, 1994, Perdew et al., 1996). Wave functions were expanded in a plane-wave basis set with a cutoff kinetic energy of 500 eV. For each computation, four formula units containing 56 atoms were constructed, and their Brillouin zone was sampled with a 3×3×3 grid based on the Monkhorst and Pack scheme (Hu et al., 2019). After geometry optimization, the formation energy of each possible structures per unit cell (Δ*E*_*f*_) (the unit cell contains four units of LZTO with periodic boundary conditions) was estimated by the following Equations: (6)

(Equation 6) $$\Delta E_{f}=\;E_{LZTO}-8\times E_{Li}-4\times E_{Zn}-12\times E_{Ti}-32\times E_{O}$$

(Equation 7) $$\Delta E_{f,\;Li↔Ta}=\;E_{LZTO,\;Li↔Ta}-7\times E_{Li}-4\times E_{Zn}-12\times E_{Ti}-32\times E_{O}-E_{Ta}$$

(Equation 8) $$\Delta E_{f,\;Zn↔Ta}=\;E_{LZTO,\;Zn↔Ta}-8\times E_{Li}-3\times E_{Zn}-12\times E_{Ti}-32\times E_{O}-E_{Ta}$$

(Equation 9) $$\Delta E_{f,\;Ti↔Mo}=\;E_{LZTO,\;Ti↔Ta}-8\times E_{Li}-4\times E_{Zn}-11\times E_{Ti}-32\times E_{O}-E_{Ta}$$

where *E*_*LZTO*_ is the total electronic energy of LZTO unit cell, and *E*_*Li*_*, E*_*Zn*_*, E*_*Ti*_*, E*_*Ta*_ *and E*_*O*_ are the total electronic energies of Li, Zn, Ti, Ta and O atoms, respectively. Li↔Ta, Zn↔Ta and Ti↔Ta refers to the substitutions of Ta for Li, Zn and Ti, respectively.

#### Characterization

The crystallographic structure and phase composition were examined by powder wide-angle X-ray diffraction (XRD) using Rigaku SmartLab SE X-ray diffractometer with a monochromated high-intensity Cu Kα radiation (*λ* = 1.5418 Å) and grazing incidence XRD (GIXRD) conducted at the X-ray diffuse scattering station on the 4W1C beamline in the Beijing Synchrotron Radiation Facility (BSRF) with the *λ* = 1.5405 Å and the energy resolution is 4.4 × 10^−4^. The *in-situ* XRD measurement was achieved by using LIB-XRD (XRD-03C, Zhongke Wanyuan Technology). Raman spectra were collected from a Horiba-Jobin Yvon LabRAM Aramis Raman system. The morphology and microstructure of Ta-doped LZTO samples were observed by JSM-7800F scanning electron microscope (SEM) with an Oxford X-MAX50 energy dispersive spectrometer (EDS) and JEOL JEM-2010 transmission electron microscope (TEM). XPS spectra were characterized on ESCALAB 250Xi (Thermo Fisher Scientific Inc., USA) with Al-K_*α*_ radiation (72 W, 12 kV) at a pressure of 10^−9^ Torr. N_2_ adsorption-desorption isotherms were obtained using a Micromeritics ASAP 2020 surface area and porosity analyzer. All samples were outgassed under vacuum for 8 h at 120°C before measurements. Brunauer-Emmett-Teller (BET) equation was used to estimate the surface area from adsorption data obtained at *P/P*_0_ = 0.01–0.30. The average pore diameter was calculated using the Barrett-Joyner-Halenda (BJH) method from the desorption branch of the isotherm. The thermal behavior was determined using thermogravimetric (TG) analysis (NETZSCH, STA 2500, Germany).

#### Electrochemical performance evaluations

LZTO-based anode materials were investigated based on the CR2032 coin-type cells at room temperature. The slurry of the working electrode was composed of 80 wt.% active materials, 10 wt.% super P, and 10 wt.% polyvinylidene fluoride (PVDF) mixing in N-Methyl-2-pyrrolidone (NMP). The slurry was then uniformly coated on Cu foil and dried in vacuum at 120°C for 12 h and rolled twice using a rolling press (MTI MSK-HRP-MR100DC). Then the foil was cut to a round disc with a diameter of 12 mm. The loading of active materials is 2.3–2.5 mg/cm^2^. The assembly of the CR2032 cell was carried out in a glove box (dry Ar atmosphere) with Celgard 2300 separator. A homogeneous 1 M LiPF_6_ solution in 1:1 ethylene carbonate (EC) and diethyl carbonate (DEC) was used as the electrolyte. All the cells were aged for 6 h before electrochemical evaluation. For the half cell, LZTO-based materials were used as the working electrode and the circular Li metal disk as the counter electrode. For the full cell, LZTO-based material and LiFePO_4_ were used as the negative and positive electrode materials, respectively. And the specific capacity is calculated based on the mass of cathode active material. All the assembled cells were charged and discharged over a voltage range of 0.05–3.0 V on a Neware battery testing system (Model: BTS-XWJ-6.44S-00052) at room temperature with diverse discharge rates from 0.1 to 3.2 A/g. CV curves were recorded on an electrochemical workstation (CHI 760E, CH Instruments) at a scanning rate of 0.5 mV/s. EIS measurements were performed on a ZIVE MP1 impedance analyzer (WonATech Corp., Korea) over the frequency of 200 kHz to 5 mHz at 5 mV as the applied sinusoidal perturbation.

## Data Availability

No new data was reported from this study. This paper does not report original code. Any additional information required to reanalyze the data reported in this paper is available from the lead contact upon request.

## Supplemental information

Document S1. Figures S1–S17, Tables S1–S 3, and Scheme S1
