# Supplementary material for: Solid-state self-template synthesis of Ta-doped Li2ZnTi3O8 spheres for efficient and durable lithium storage
Source: iScience. 2021 Aug 18;24(9):102991. doi: 10.1016/j.isci.2021.102991 (PMC8405915; doi:10.1016/j.isci.2021.102991)
Supplement: Document S1. Figures S1–S17, Tables S1–S — 3, and Scheme S1 [file mmc1.pdf]

iScience, Volume *24*

Supplemental information

Solid-state self-template synthesis of Ta-doped $Li_2ZnTi_3O_8$ spheres for efficient and durable lithium storage

Dongwei Ma, Jiahui Li, Jing Yang, Chengfu Yang, Maykel Manawan, Yongri Liang, Ting Feng, Yong-Wei Zhang, and Jia Hong Pan

Supplemental Figures

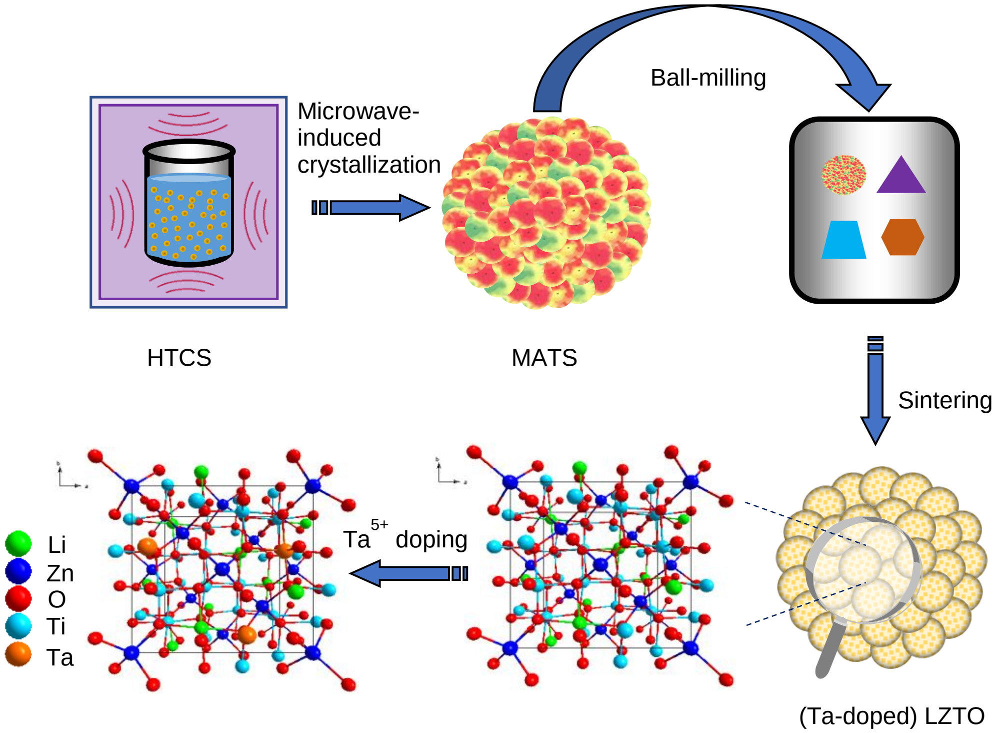

Scheme S1. Schematic illustration of the self-template synthesis of MATS via the microwave-induced crystallization of amorphous HTCS, and their subsequent solid-state self-template synthesis of Ta-doped LZTO spheres. Related to STAR Methods.

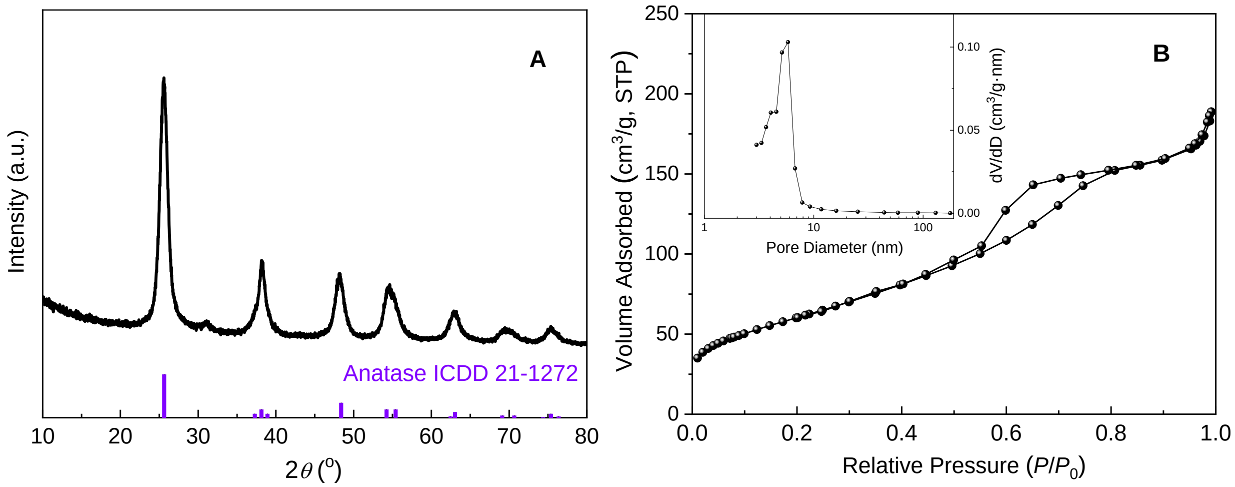

Figure S1. XRD patterns, N_2 sorption isotherms, and the corresponding pore size distributions (inset) of MATS. Related to Figure 1.

(A) XRD patterns, (B) N_2 sorption isotherms and the corresponding pore size distributions (inset) of MATS, the self-template for the subsequent solid-state synthesis of (Ta-doped) LZTO spheres.

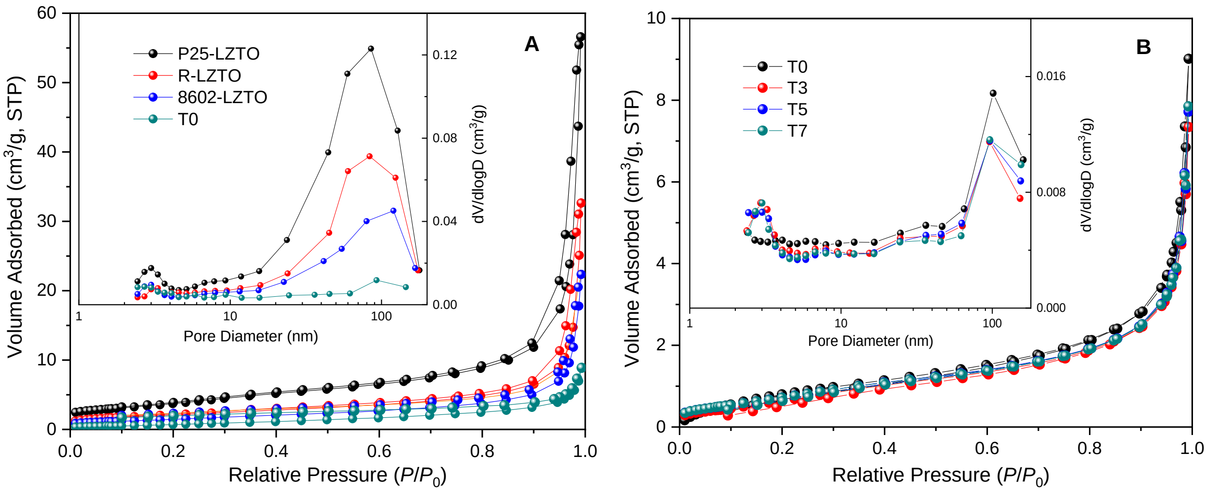

Figure S2. N$_2$ sorption analyses of LZTO from different TiO$_2$ sources and Ta-doped LZTO spheres. Related to Figure 2.

N$_2$ sorption isotherms and the pore size distributions (inset) of (A) LZTO with different TiO$_2$ sources and (B) LZTO spheres doped with different amounts of Ta^{5+} ions.

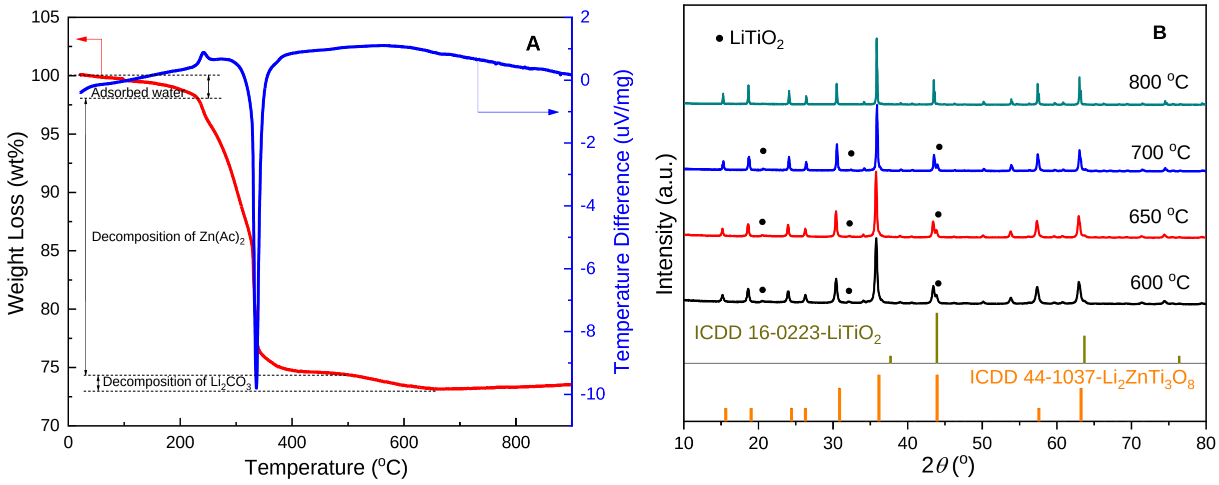

Figure S3. TG-DTA curves and XRD patterns of T0. Related to Figure 2.

(A) TG–DTA curves of the ball-milling solid precursor for T0, and (B) XRD patterns of T0 synthesized at different calcination temperatures.

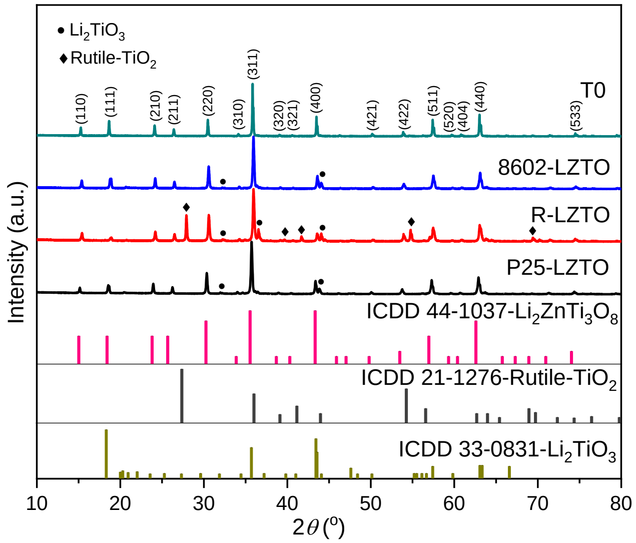

Figure S4. XRD patterns of P25-LZTO, R-LZTO, 8602-LZTO, and T0. Related to Figure 2.

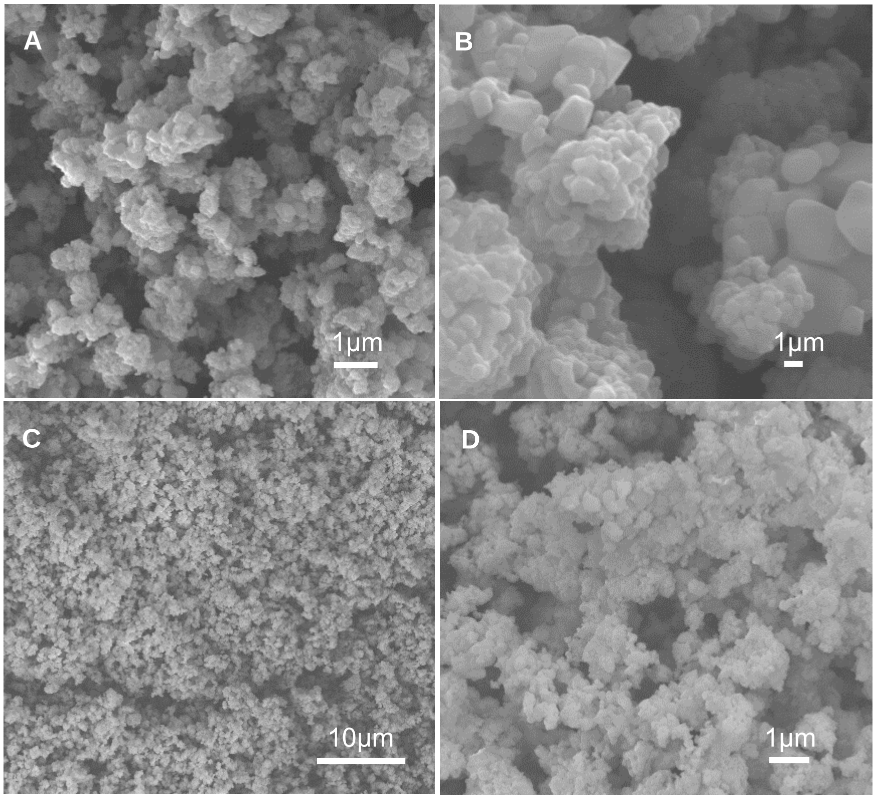

Figure S5. SEM images of P25-LZTO and 8602-LZTO. Related to Figure 2.

SEM images of (A, B) P25-LZTO and (C, D) 8602-LZTO derived from different TiO_2 nanoparticles of Aeroxide P25 and Hombikat 8602, respectively.

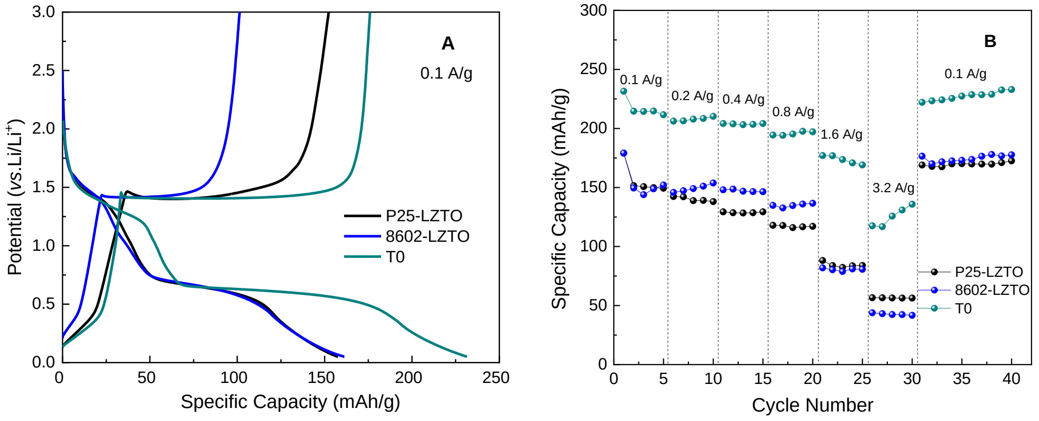

Figure S6. Initial charge/discharge curves and rate performance of LZTO with different TiO_2 sources. Related to Figure 7.

(A) Initial charge/discharge curves at 0.1 A/g and (B) Rate performance of T0, P25-LZTO and 8602-LZTO working in the potential range of 0.05–3.0 V.

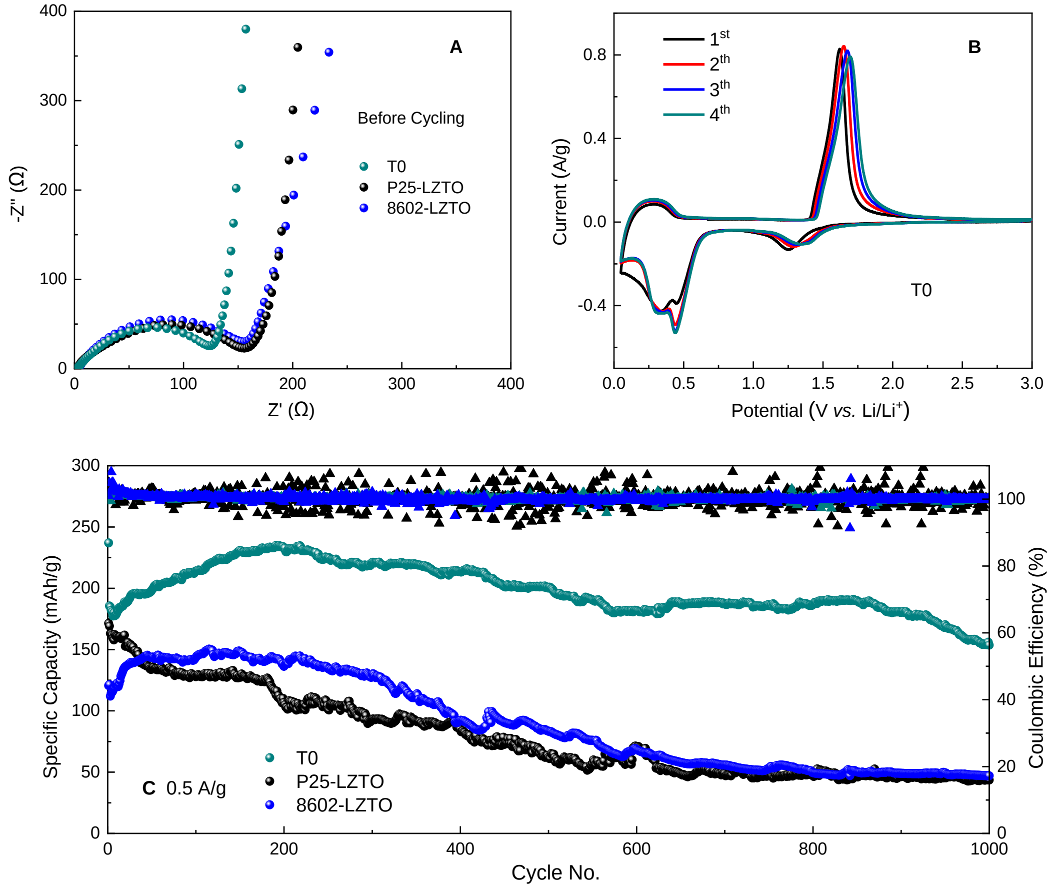

Figure S7. Comparative EIS spectra, CV curves, and cycling performances of LZTO from different TiO_2 sources. Related to Figure 7.

(A) Open-circuit EIS spectra, (B) CV plots from 1st to 4th cycle at 0.5 mV/s (voltage range: 0.05-3.0 V), and (C) cycling performances and Coulombic efficiencies at 0.5 A/g of T0, P25-LZTO and 8602-LZTO working in the potential range of 0.05–3.0 V.

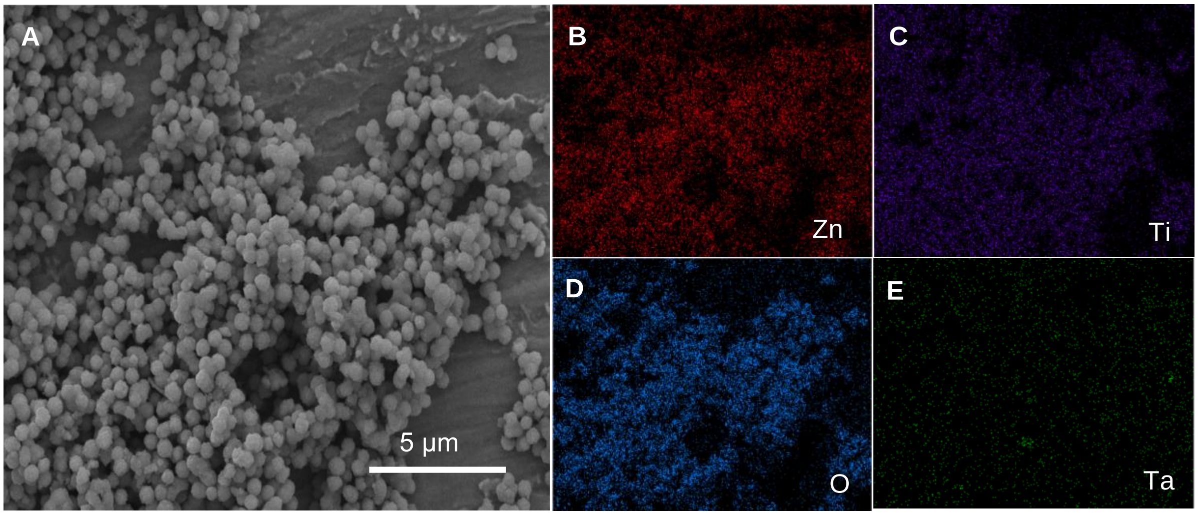

Figure S8. SEM image and EDS spectra of T5. Related to Figure 2.

(A) Representative SEM image of T5 and EDS spectra of (B) Zn, (C) Ti, (D) O and (E) Ta elements.

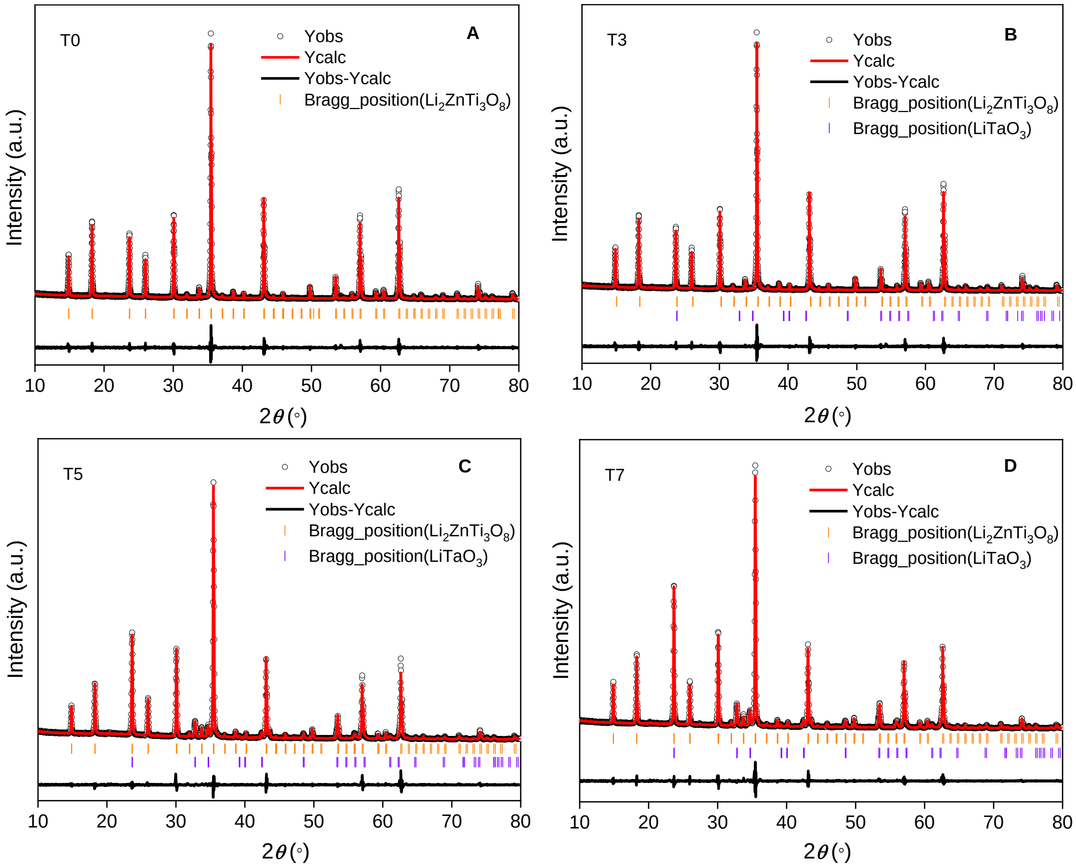

Figure S9. Rietveld refinements for XRD patterns. Related to Figure 3.

Rietveld refinements for XRD patterns of (A) T0, (B) T3, (C) T5 and (D) T7 using TOPAS program.

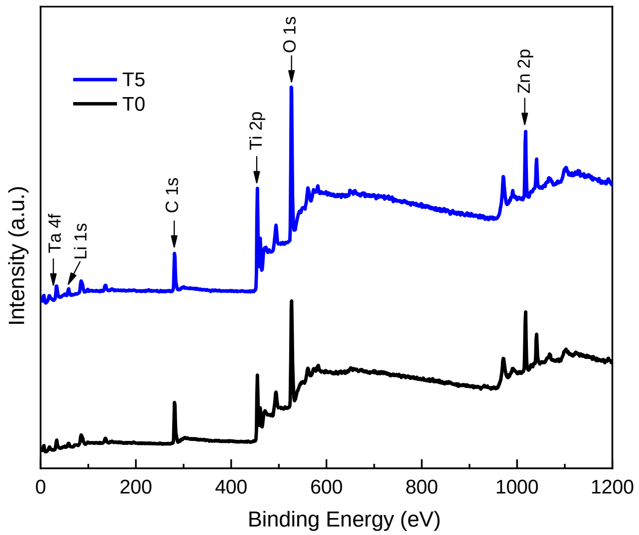

Figure S10. XPS survey spectra of T0 and T5. Related to Figure 3.

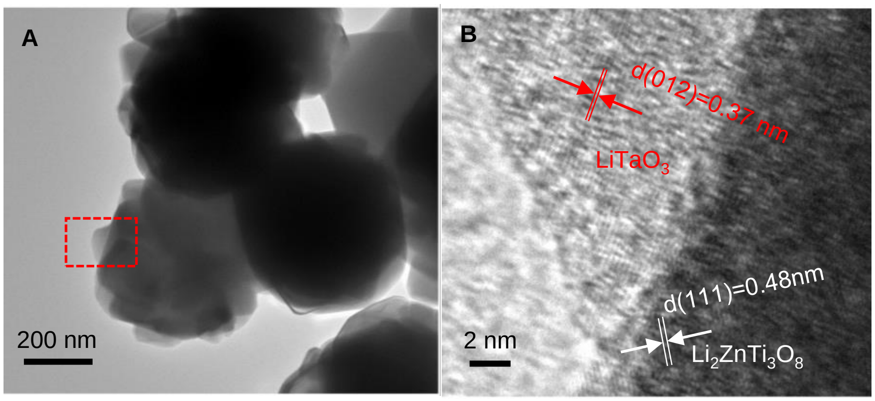

Figure S11. TEM images of T5. Related to Figure 4.

(A) TEM and (B) high-resolution TEM images of T5.

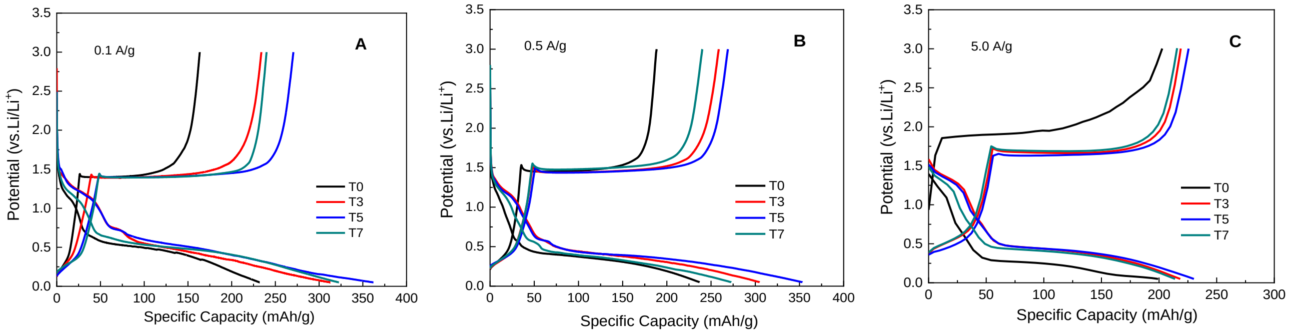

Figure S12. Initial charge/discharge curves at different current densities. Related to Figure7.

Initial charge/discharge curves of T0, T3, T5, and T7 at different current densities of (A) 0.1 A/g, (B) 0.5 A/g, and (C) 5.0 A/g.

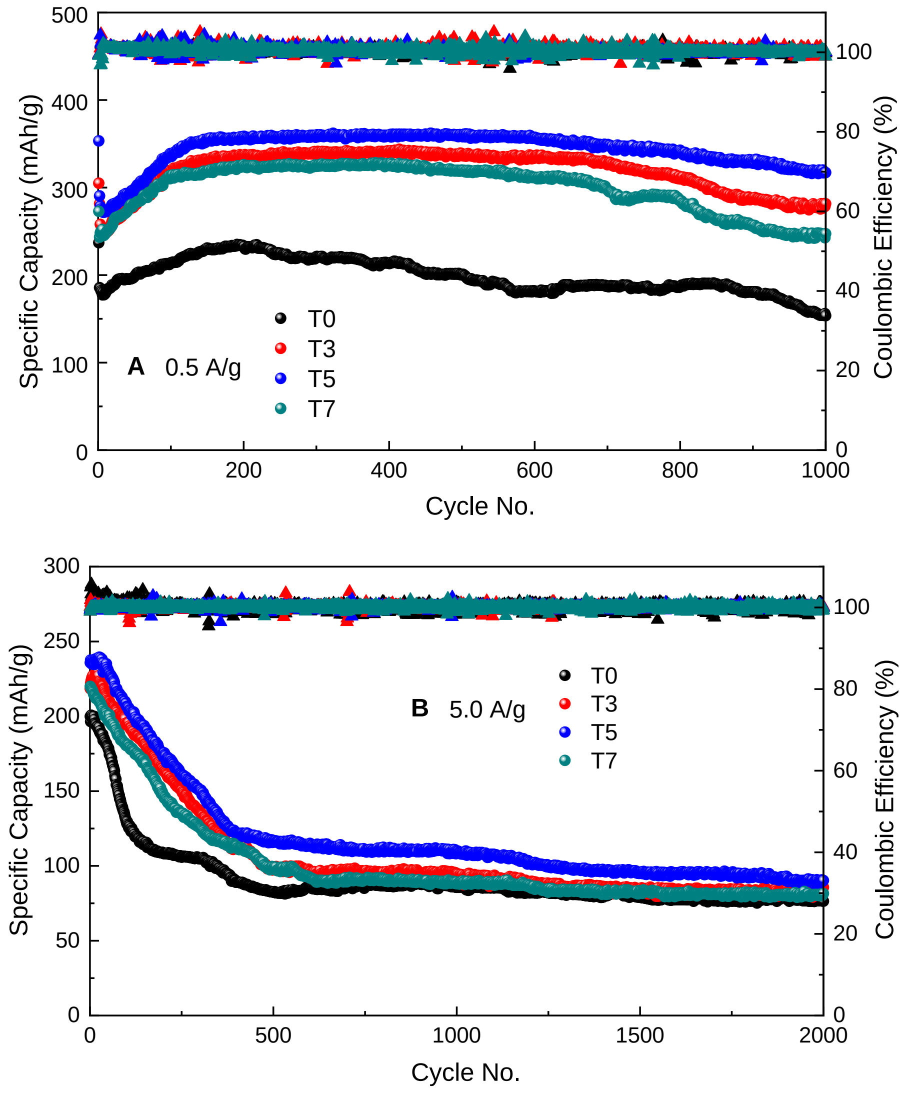

Figure S13. Cycling performances and Coulombic efficiencies at different current densities. Related to Figure 7.

Cycling performances and Coulombic efficiencies of T0, T3, T5, and T7 in 0.05–3.0 V at different current densities of (A) 0.5 A/g and (B) 5.0 A/g.

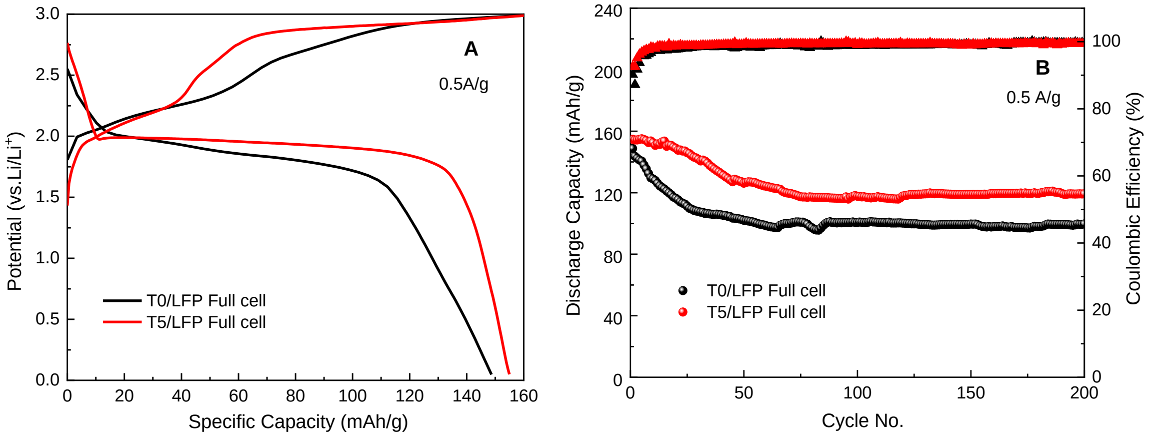

Figure S14. Electrochemical performances of full cells. Related to Figure 7.

(A) Initial charge-discharge curves and (B) cycle performances of T0/$LiFePO_4$ and T5/$LiFePO_4$ full cell at a current density of 0.5 A/g.

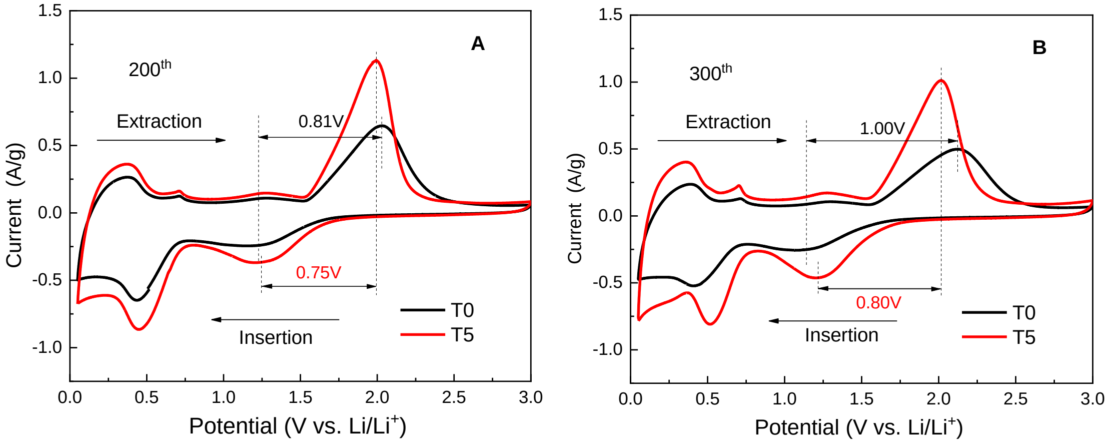

Figure S15. CV plots of T0 and T5. Related to Figure 8.

CV plots of T0 and T5 in the voltage range of 0.05–3.0 V at (A) 200th and (B) 300th cycles at a scanning rate of 0.5 mV/s.

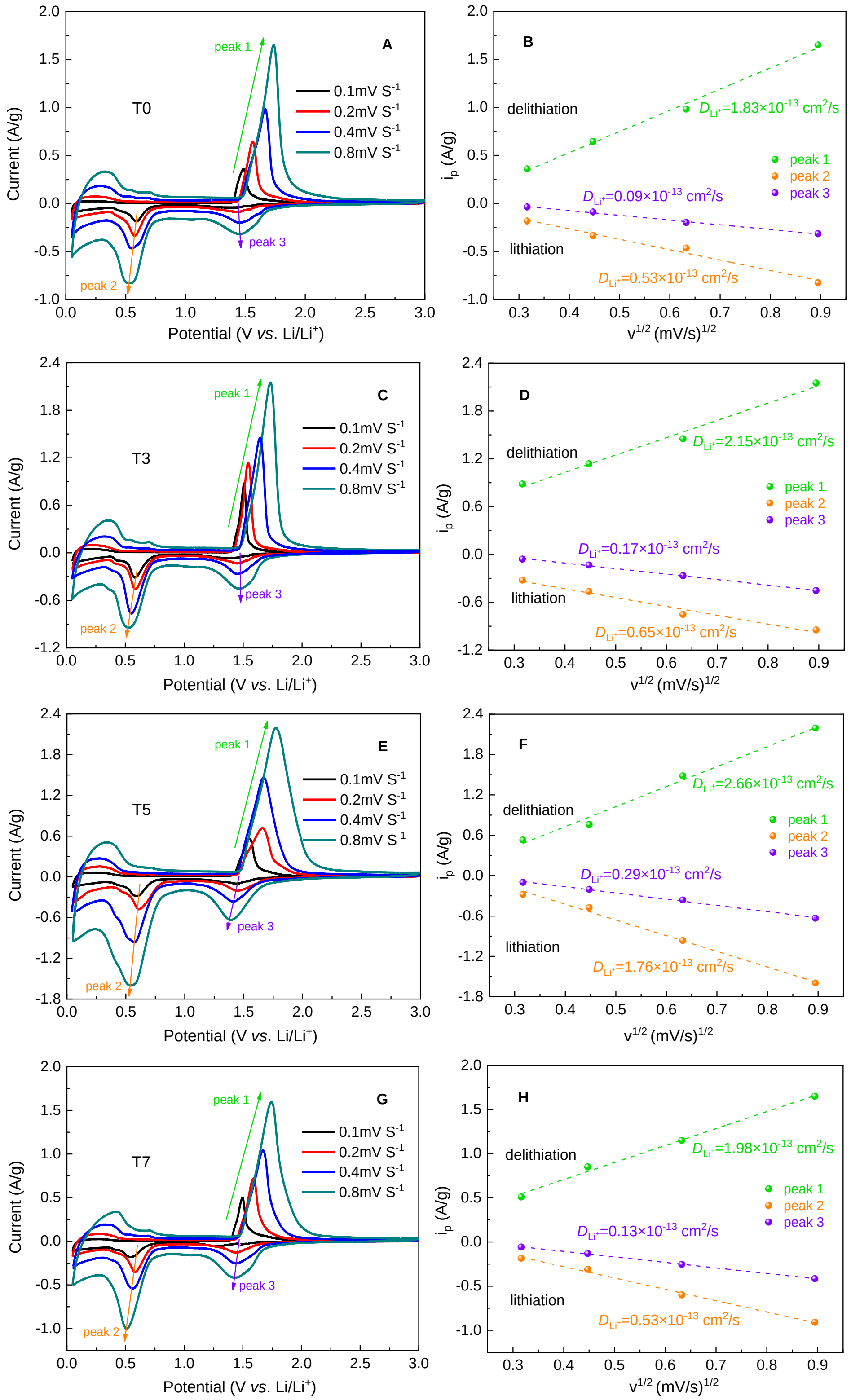

Figure S16. CV curves and corresponding relationship between peak current density of cathodic/anodic reaction (i_p) and square root of scan speed ($v^{0.5}$). Related to Figure 8.

CV curves under various scan rates in half cells, and corresponding relationship between i_p and $v^{0.5}$ for (A, B) T0, (C, D) T3, (E, F) T5 and (G, H) T7.

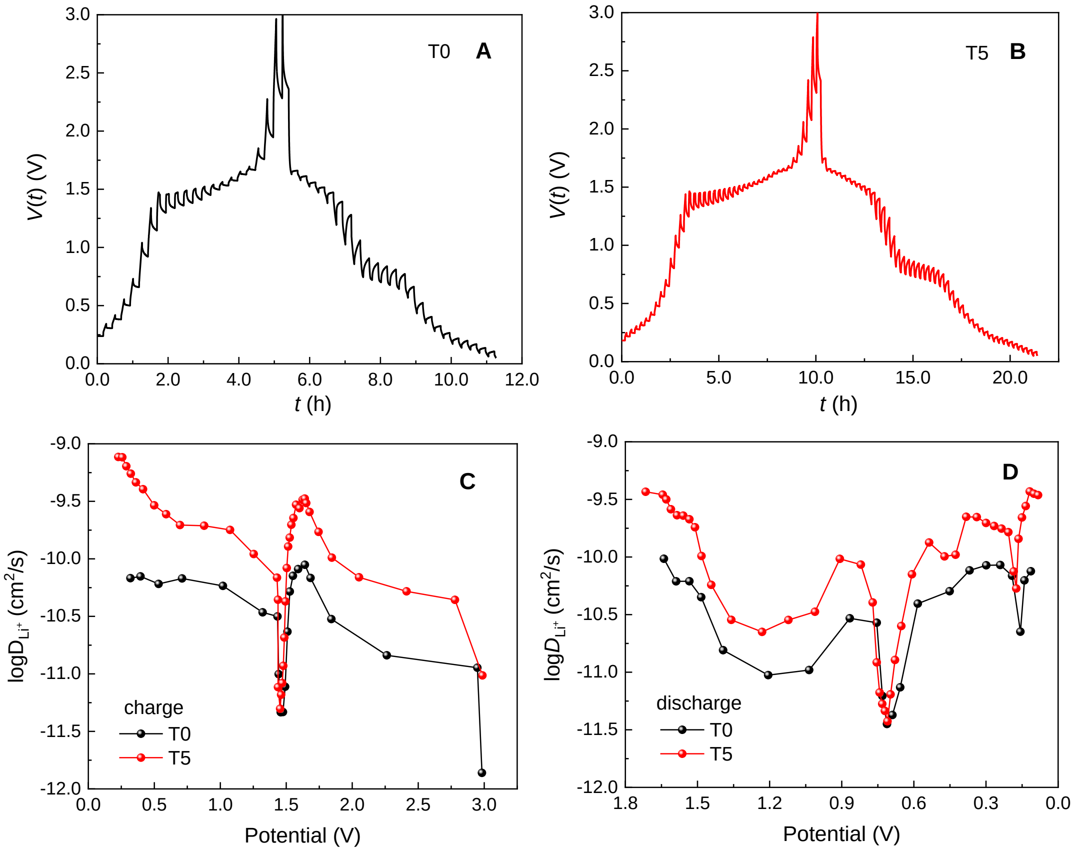

Figure S17. GITT curves for T0 and T5. Related to Figure 8.

Charge/discharge GITT curves for (A) T0 and (B) T5 at 100 mA/g; Corresponding Li^+ coefficients during the 100th (C) charge and (D) discharge processes at room temperature for T0 and T5.

Supplemental Tables

Table S1. BET surface area of different LZTO samples calculated from their N_2 sorption isotherms in Figure S5. Related to Figure 2 and STAR Methods.

Sample	BET surface area (m^2/g)
P25-LZTO	13.75
R-LZTO	7.72
8602-LZTO	5.49
T0	3.00
T3	2.93
T5	2.95
T7	2.93

Table S2. TOPAS evaluation parameters and lattice parameters of T0, T3, T5 and T7. Here R_p is profile residual (Unweighted); R_{wp} is weighted profile *R*-factor; and *V* is lattice volume. Related to Figure 3.

Sample	R_p (%)	R_{wp} (%)	$a = b = c$ (Å)	V (Å^3)	$LiTaO_3$ (wt%)	doped Ta^{5+} (at%)
T0	2.65	9.02	8.3712	586.5786	--	--
T3	6.80	8.77	8.3745	587.3180	1.21	2.49
T5	2.32	10.80	8.3761	587.6580	3.50	3.52
T7	5.95	7.81	8.3773	587.9130	4.17	5.23

Table S3. Comparison of textural properties of T5 and its specific capacities after cycling with the reported values in literature delivered by various LZTO. Related to Figure 7 and STAR Methods.

Cathode material	Synthetic method	Average particle size (nm)	First-cycle capacity (mAh/g)	Last-cycle capacity (mAh/g after cycles)	Current Density	Ref.
LZTO nanoparticles	microwave	45	175	208 after 25 cycles	0.5 A/g	(Li et al., 2016)
LZTO/C nanoparticles	sol-gel	25	408	284 after 200 cycles	0.2 A/g	(Xu et al., 2013)
LZTO/C nanobelts	sol-gel	75 (diameter)	374	248 after 200 cycles	0.2 A/g	(Lan et al., 2017)
$Li_2Zn_{0.93}Mo_{0.07}Ti_3O_8$ /GR nanoparticles	solid-state	30	285	240 after 600cycles	1.0 A/g	(Wang et al., 2019)
LZTO nanofibers	electrospinning	200 (diameter)	370	214 after 40 cycles	0.2 A/g	(Wang et al., 2011)
LZTO nanoparticles	molten-salt	100	168	138 after 100 cycles	2.0 A/g	(Chen et al., 2015)
Mg^{2+}-W^{6+} co-doped LZTO nanoparticles	solid-state	27	220	208 after 400 cycles	1.0 A/g	(Shen et al., 2019)
$Li_2ZnTi_{2.95}Nb_{0.05}O_8$ nanoparticles	solid-state	100	310	215 after 40 cycles	0.2 A/g	(Firdous et al., 2020)
La_2O_3@LZTO/C nanoparticles	sol-gel	35	347	250 after 100 cycles	1.0 A/g	(Meng et al., 2019)
Lithium magnesium silicate@LZTO nanoparticles	solid-state	200	236	190 after 400 cycles	0.5 A/g	(Yang et al., 2019)
LZTO/N-doped C nanoparticles	solid-state	70	227	194 at 500 cycles	1.0 A/g	(Tang et al., 2020)
$Li_2ZnTi_{2.95}Ce_{0.05}O_8$ nanoparticles	solid-state	200	197	133 after 500 cycles	2.0 A/g	(Chen et al., 2017)
**T5**	**self-template solid-state**	**450**	**230**	**195 after 1000 cycles**	**1.0 A/g**	**This work**

Supplemental References

Chen, B., Du, C., Zhang, Y., Sun, R., Zhou, L., Wang, L., 2015. A new strategy for synthesis of lithium zinc titanate as an anode material for lithium ion batteries. Electrochim. Acta 159, 102–110.

Chen, C., Ai, C., Liu, X., Wu, Y., 2017. Advanced electrochemical properties of Ce-modified $Li_2ZnTi_3O_8$ anode material for lithium-ion batteries. Electrochim. Acta 227, 285–293.

Firdous, N., Arshad, N., Simonsen, S.B., Kadirvelayutham, P., Norby, P., 2020. Advanced electrochemical investigations of niobium modified $Li_2ZnTi_3O_8$ lithium ion battery anode materials. J. Power Sources 462, 228186.

Li, Z.F., Cui, Y.H., Wu, J.W., Du, C.Q., Zhang, X.H., Tang, Z.Y., 2016. Synthesis and electrochemical properties of lithium zinc titanate as an anode material for lithium ion batteries via microwave method. RSC Adv. 6, 39209–39215.

Lan, T., Chen, L., Liu, Y., Zhang, W., Wei, M., 2017. Nanocomposite $Li_2ZnTi_3O_8$/C with enhanced electrochemical performances for lithium-ion batteries. J. Electroanal. Chem. 794, 120–125.

Meng, Z., Wang, Suhong, Wang, H., Wang, L., Wang, Song, 2019. La_2O_3-coated $Li_2ZnTi_3O_8$@C as a high performance anode for lithium-ion batteries. RSC Adv. 9, 20618–20623.

Shen, Z., Zhang, Z., Wang, S., Liu, Z., Wang, L., Bi, Y., Meng, Z., 2019. Mg^{2+}-W^{6+} co-doped $Li_2ZnTi_3O_8$ anode with outstanding room, high and low temperature electrochemical performance for lithium-ion batteries. Inorg. Chem. Front. 6, 3288–3294.

Tang, H., Chen, C., Liu, T., Tang, Z., 2020. Chitosan and chitosan oligosaccharide: Advanced carbon sources are used for preparation of N-doped carbon-coated $Li_2ZnTi_3O_8$ anode material. J. Electroanal. Chem. 858, 113789.

Wang, S., Bi, Y., Wang, L., Meng, Z., Luo, B., 2019. Mo-doped $Li_2ZnTi_3O_8$ @graphene as a high performance anode material for lithium-ion batteries. Electrochim. Acta 301, 319–324.

Wang, L., Wu, L., Li, Z., Lei, G., Xiao, Q., Zhang, P., 2011. Synthesis and electrochemical properties of $Li_2ZnTi_3O_8$ fibers as an anode material for lithium-ion batteries. Electrochim. Acta 56, 5343–5346.

Xu, Y., Hong, Z., Xia, L., Yang, J., Wei, M., 2013. One step sol-gel synthesis of $Li_2ZnTi_3O_8$/C nanocomposite with enhanced lithium-ion storage properties. Electrochim. Acta 88, 74–78.

Yang, H., Lun, N., Qi, Y.X., Zhu, H.L., Liu, J.R., Feng, J.K., Zhao, L. ling, Bai, Y.J., 2019. $Li_2ZnTi_3O_8$ coated with uniform lithium magnesium silicate layer revealing enhanced rate capability as anode material for Li-Ion battery. Electrochim. Acta 315, 24–32.
